# Impact of SIRPα genotype combinations in recipients and donors on alloimmune response in liver transplantation

**DOI:** 10.1093/pnasnexus/pgaf351

**Published:** 2025-11-05

**Authors:** Akhmet Seidakhmetov, Naoki Tanimine, Yuka Tanaka, Ryosuke Arata, Ryosuke Nakano, Hiroshi Sakai, Masahiro Ohira, Hiroyuki Tahara, Kentaro Ide, Tsuyoshi Kobayashi, Hideki Ohdan

**Affiliations:** Department of Gastroenterological and Transplant Surgery, Graduate School of Biomedical and Health Sciences, Hiroshima University, 1-2-3 Kasumi, Minami-ku, Hiroshima 734-8551, Japan; Department of Gastroenterological and Transplant Surgery, Graduate School of Biomedical and Health Sciences, Hiroshima University, 1-2-3 Kasumi, Minami-ku, Hiroshima 734-8551, Japan; Department of Surgery, Kure Medical Center, Chugoku Cancer Center, 3-1 Aoyama-cho, Kure, Hiroshima 737-0023, Japan; Department of Gastroenterological and Transplant Surgery, Graduate School of Biomedical and Health Sciences, Hiroshima University, 1-2-3 Kasumi, Minami-ku, Hiroshima 734-8551, Japan; Department of Gastroenterological and Transplant Surgery, Graduate School of Biomedical and Health Sciences, Hiroshima University, 1-2-3 Kasumi, Minami-ku, Hiroshima 734-8551, Japan; Department of Gastroenterological and Transplant Surgery, Graduate School of Biomedical and Health Sciences, Hiroshima University, 1-2-3 Kasumi, Minami-ku, Hiroshima 734-8551, Japan; Department of Gastroenterological and Transplant Surgery, Graduate School of Biomedical and Health Sciences, Hiroshima University, 1-2-3 Kasumi, Minami-ku, Hiroshima 734-8551, Japan; Department of Gastroenterological and Transplant Surgery, Graduate School of Biomedical and Health Sciences, Hiroshima University, 1-2-3 Kasumi, Minami-ku, Hiroshima 734-8551, Japan; Department of Gastroenterological and Transplant Surgery, Graduate School of Biomedical and Health Sciences, Hiroshima University, 1-2-3 Kasumi, Minami-ku, Hiroshima 734-8551, Japan; Department of Gastroenterological and Transplant Surgery, Graduate School of Biomedical and Health Sciences, Hiroshima University, 1-2-3 Kasumi, Minami-ku, Hiroshima 734-8551, Japan; Department of Gastroenterological and Transplant Surgery, Graduate School of Biomedical and Health Sciences, Hiroshima University, 1-2-3 Kasumi, Minami-ku, Hiroshima 734-8551, Japan; Department of Gastroenterological and Transplant Surgery, Graduate School of Biomedical and Health Sciences, Hiroshima University, 1-2-3 Kasumi, Minami-ku, Hiroshima 734-8551, Japan

**Keywords:** alloimmune response, polymorphism, signal regulatory protein alpha (SIRPα), transplantation

## Abstract

The signal regulatory protein alpha (SIRPα)–CD47 axis regulates self-tolerance by delivering inhibitory signals to phagocytic cells and influencing immune responses in transplantation. This study examined the impact of SIRPα polymorphisms on CD47 binding capacity and T cell activation and analyzed the role of SIRPα genotypes in acute rejection following allogeneic liver transplantation. Peripheral blood mononuclear cells from healthy volunteers and data from liver transplant recipients were analyzed. Results showed that V2 SIRPα exhibited significantly higher CD47 binding capacity and enhanced costimulatory activity on CD4^+^ T cell proliferation compared V1 under both CD28^−^ and CD28^+^ conditions, with further augmentation in the CD28^+^ setting. In the liver transplant cohort, SIRPα V2 was associated with higher acute rejection incidences than V1. These findings suggest that SIRPα polymorphisms, particularly V2, enhance T cell activation and modulate alloimmune responses through both innate and adaptive immunity, with potential implications for transplant outcomes. SIRPα genotyping may serve as one component within broader, multiparameter risk models for acute rejection and help inform optimization of immunosuppressive strategies.

Significance Statement This study is the first to elucidate the clinical significance of donor-recipient signal regulatory protein alpha (SIRPα) genotype combinations in transplantation immunology. Specific SIRPα polymorphisms not only modulate innate immune responses but also influence acute rejection outcomes through acquired immune response in liver transplantation. This work provides compelling mechanistic evidence that SIRPα genotyping can contribute as one component of broader, multiparameter risk models, advancing precision medicine and transplant immunobiology.

## Introduction

The signal regulatory protein alpha (SIRPα) was revealed as a SH2-domain-containing phosphotyrosine phosphatase-binding protein peptide sequenced with complementary DNA cloning (1). A previous study that cloned human SIRPα reported its extracellular part to be polymorphic (2). SIRPα and its corresponding ligand, integrin-associated protein (CD47), form a self-tolerance mechanism through the “don’t eat me signal” for phagocytotic myeloid cells (monocytes, macrophages, and dendritic cells) regulating innate immune response (3). We have previously shown that the interspecies incompatibility of CD47 is responsible for rejection mediated by macrophage phagocytosis in xenogeneic transplantation (4). During the same period, the impact of SIRPα polymorphism on the engraftment of hematopoietic stem cell transplantation was demonstrated in a xenogeneic hematopoietic stem cell transplantation model with immunodeficient animals lacking acquired immunity (5). These findings highlight that in xenotransplantation, the regulatory inhibitory signals mediated by SIRPα in host immune cells, particularly macrophages and dendritic cells, are unable to function effectively. This dysfunction underpins the distinct mechanisms of rejection that are characteristic of xenografts. Although similar pathways are generally not considered to drive rejection during allogeneic transplantation, a recently study has shown that the SIRPα polymorphism in a mouse allogeneic transplantation model affects the binding capacity for CD47 and induces major histocompatibility complex-independent activation of SIRPα-bearing cells through a bidirectional signaling balance involving the SIRPα-CD47 interaction (6). This discovery builds upon previous findings that the CD47-SIRPα pathway functions as a bidirectional signaling mechanism, transmitting inhibitory signals to SIRPα-expressing immune cells while conveying activation signals to CD47-expressing cells (7, 8). Donors that differed from the recipient in one or both SIRPα alleles elicited an alloresponse of monocytes, indicating that sensing SIRPα polymorphism by CD47 activates a molecular mechanism by which the innate immune system distinguishes between self and allogeneic non-self independently of T and B cells. Since SIRPα and CD47 are coexpressed on monocytes, transplantation of allogeneic grafts carrying non-self SIRPα alleles likely activates innate immunity by integrating activating and inhibitory signals delivered by CD47 and SIRPα, respectively.

Beyond innate immunity, activation signals from SIRPα to CD47 have been shown to enhance immune responses in T cells stimulated by anti-CD3 agonist antibodies (Abs) (9–11). This immune modification, which coincides with polyclonal stimulation via T cell receptor (TCR) signaling, is similar to activation by classical costimulatory molecules, such as CD28 and 4-1BB, and activates T cells through several mechanisms, including increased cytokine production and proliferation. Therefore, the SIRPα–CD47 axis may be involved not only in innate immune regulation but also in the regulation of adaptive immune responses. However, during T cell activation involving antigen-presenting cells (APCs) from both donor and recipient, the precise role of SIRPα in modulating the CD47 signal on T cell activation remains unclear and warrants further investigation.

In this study, we elucidated the impact of the SIRPα genotype on CD47-binding capacity and costimulatory effects on T cells in vitro. Additionally, we investigated the effect of recipient and donor SIRPα polymorphism combinations on rejection in a liver transplant cohort.

## Results

### Genotyping SIRPα IgV domain polymorphism through SNP-based recognition

The reported polymorphic SIRPα IgV domain variants can be recognized as haplotypes consisted of 15 responsible single nucleotide polymorphisms (SNPs) and 1 deletion, rs555038801, rs755247899, rs143735290, rs17855609, rs17855610, rs17855611, rs17855612, rs1057114, rs138283486, rs149634649, rs386811662, rs17855615, rs17855616 rs139878822, and rs114499682 and rs561231326 (Fig. S1).

First, 94 DNA samples from healthy volunteers were genotyped for the SIRPα IgV domain using Sanger sequencing. We identified 12 (12.8%) and 35 (37.2%) individuals with the V1/V1 and V2/V2 homozygous genotypes, respectively. However, the heterozygous genotype could not be accurately determined in 47 individuals (50%) using Sanger sequencing (Fig. S2). We intentionally use the peripheral blood mononuclear cells (PBMCs) from the healthy volunteers characterized as either V1/V1 or V2/V2 for in vitro assay to investigate the impact of the SIRPα genotype (Table S1).

**Fig. 1.**
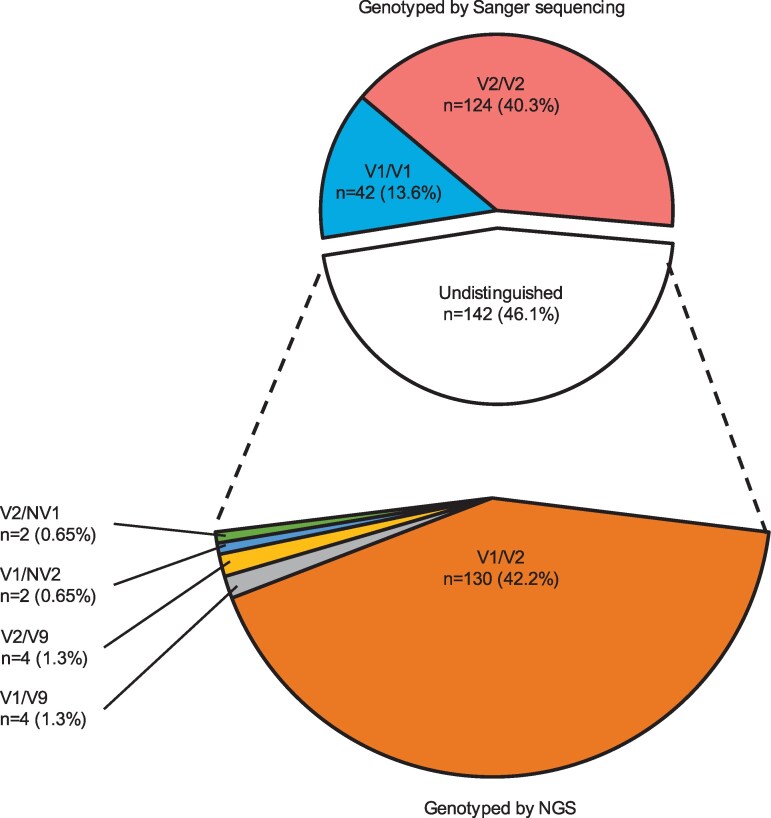
Comprehensive genotyping for SIRPα in 154 recipient and donor pairs of living donor liver transplantation cohort. Homogenous genotypes (V1/V1 and V2/V2) were identified using Sanger sequencing. Samples undistinguished (*n* = 142) by Sanger sequencing were genotyped by next-generation sequencing (NGS), which revealed a majority of heterogeneous V1/V2 with a small proportion of V9 and new variants (NV1 and 2).

Next, using Sanger sequencing, 308 DNA samples from 154 pairs of donors and recipients who underwent living-donor liver transplantation (LT) were genotyped for the SIRPα IgV domain. The V1/V1, V2/V2, and undistinguished heterozygous genotypes were similarly distributed in the healthy volunteer cohort (13.6, 40.3, and 46.1%, respectively). Therefore, we performed next-generation sequencing (NGS) analysis on 142 undistinguished individuals and found 5 different haplotypes, including two new haplotypes in our Japanese cohort (V1, V2, V9, new variant 1 [NV1], and new variant 2 [NV2]) (Figs. 1 and S3). The allele frequencies of V1, V2, V9, NV1, and NV2 were 35.7, 62.4, 1.3, 0.3, and 0.3%, respectively, comparable between the recipients and donors (Table S2). Additionally, allele frequency was not associated with primary disease in the recipient (Table S3). We observed a similarity in the SNP repertoire in V9, NV1, and NV2 compared with the V1 repertoire (Fig. S3).

### V2 SIRPα on monocytes showed higher binding capacity to CD47 than V1 SIRPα

**Fig. 2.**
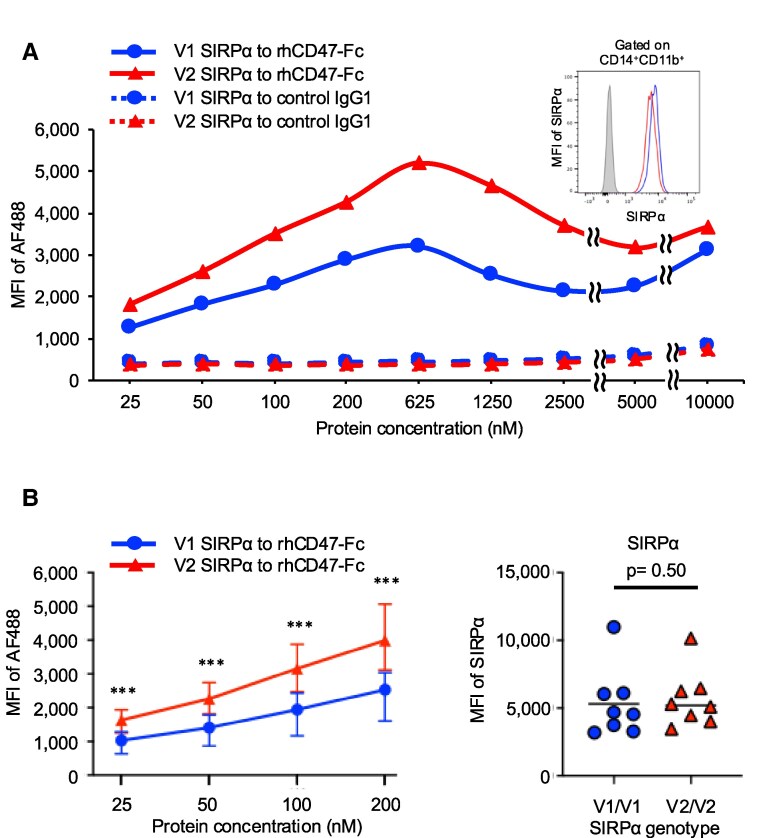
V2 SIRPα on human monocytes showed higher binding capacity with recombinant human CD47-Fc protein. PBMCs from healthy volunteers (V1/V1, circle or V2/V2, triangle) were incubated with Alexa Fluor 488 binding recombinant human (rh) CD47-Fc protein. MFI of Alexa Fluor 488 was evaluated as binding capacity of SIRPα on monocytes (gated on CD14^+^CD11b^+^) with CD47 protein. A) Representative plots showing the kinetic binding capacity according to CD47 concentration. The upper-right histogram shows the corresponding SIRPα expression on monocytes. B) The SIRPα V2 variant (*n* = 8) exhibits higher binding capacity than V1 (*n* = 8). Data are presented as medians (central bars) with ranges (error bars). Dot plots depict the mean fluorescence intensity (MFI) of SIRPα on monocytes from V1/V1 and V2/V2 volunteers; Statistical analyses were performed using Mann–Whitney *U* test. SIRPα, signal-regulatory protein alpha; IgG1, immunoglobulin G1; rhCD47, recombinant human CD47; MFI, mean fluorescent intensity; *** *P* < 0.001.

The expression levels of SIRPα on CD14^+^CD11b^+^ monocytes were not correlated with the SIRPα genotype (Fig. S4). We assessed the binding capacity of SIRPα on monocytes from V1/V1 or V2/V2 variants to recombinant human CD47 (rhCD47)-Fc protein, as measured by MFI of AF488. SIRPα on V2/V2 variant monocytes shows a higher binding response to rhCD47-Fc protein across most concentrations compared with V1/V1 monocytes, despite slightly higher SIRPα expression on V1/V1 monocytes, no binding response was detected with the control IgG1 lacking the CD47 in either variant (Fig. 2A). In the dose-dependent concentration range of CD47-Fc (25, 50, 100, and 200 nM), monocytes with the V2/V2 variant consistently showed a higher binding affinity than monocytes with the V1/V1 variant. The MFI of SIRPα on monocytes was comparable between V1/V1 and V2/V2 variants (Fig. 2B). These data suggest that SIRPα in V2 variants SIRPα exhibits a greater binding capacity/affinity for CD47 than SIRPα in V1 variants.

Furthermore, to assess the impact of inflammatory conditions, we examined binding after lipopolysaccharide (LPS) preactivation. Consistent with prior reports (12), SIRPα expression on monocytes was downregulated after 3 days of LPS exposure; however, the V2 variant consistently exhibited greater CD47 binding than the V1 variant in SIRPα⁺ monocytes (Fig. S5).

### V2 SIRPα enhanced proliferation of CD4+ effector T cells under TCR stimuli

**Fig. 3.**
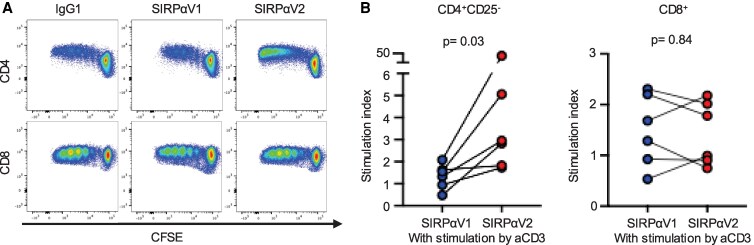
SIRPα-Fc V2 protein significantly enhanced proliferation of effector CD4+ T cells under TCR stimulation compared with SIRPα-Fc V1 protein. CD4+CD25− or CD8+ T cells isolated using FACS-sorting from healthy volunteers PBMCs labeled with CFSE were cultured for five days with immobilized anti-CD3 Ab (5 μg/mL), with IgG1 control Ab, SIRPα V1, or V2-Fc protein (5 μg/mL). A) Representative flow plots showing the proliferation of CD4+ effector (upper panel) and CD8+ (lower panel) T cells. All the other flow plots are shown in Fig. S6. B) Dot plots showing the stimulation indices of CD4+ and CD8+ T cells from three separate experiments (*n* = 6). Statistical analyses were performed using the Wilcoxon matched-pairs signed rank test. *P* < 0.05 was considered significant. SIRPα, signal-regulatory protein alpha; IgG1, immunoglobulin G1; CFSE, carboxyfluorescein diacetate succinimidyl ester.

Costimulatory signaling from SIRPα to CD47 has been demonstrated to enhance T cell responses stimulated by anti-CD3 agonist antibodies (9–11). This fact raises the question of whether the SIRPα genotype influences the extent of CD47-mediated costimulatory signaling in T cells stimulated by anti-CD3 Abs. T cells isolated from the PBMCs of six volunteers were cultivated in plates coated by anti-CD3 Ab with either human SIRPα-Fc recombinant protein V1 genotype or V2 genotype. The proliferation of CD4^+^ T cells conditioned with V2 SIRPα-Fc was significantly enhanced compared with that with V1 (median SI of V1 1.44 vs. V2 2.89, *P* < 0.03, Figs. 3 and S6). The effect of V2 SIRPα on CD4⁺ T cells was reproducible (Fig. S7) and consistently observed in both naïve and effector-memory CD4⁺ T cells (Fig. S8). The proliferation of CD8^+^ T cells was not affected by the SIRPα genotype (median SI of V1 1.49 vs. V2 1.39, *P* = 0.84). The data demonstrate that the SIRPα V2 protein induces significantly stronger costimulatory signaling in effector CD4^+^ T cells compared with the V1 protein, which is consistent with the stronger binding capacity of SIRPα V2 to CD47 relative to V1.

**Fig. 4.**
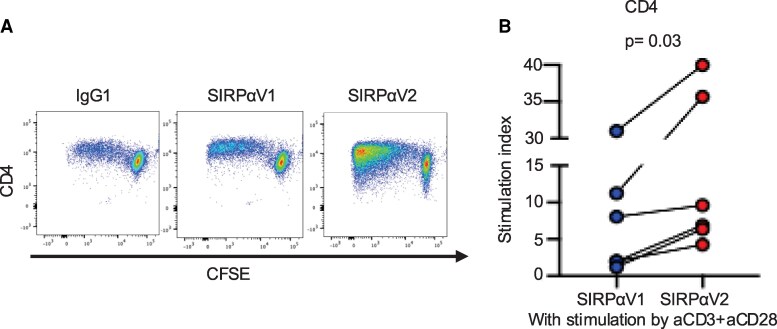
Compared with SIRPα-Fc V1, SIRPα-Fc V2 potentiates CD28 costimulation during TCR engagement. CFSE-labeled CD4⁺CD25⁻ T cells were cultured for 5 days with plate-bound anti-CD3 (5 μg/mL) and anti-CD28 (10 μg/mL) in the presence of an IgG1 isotype control, SIRPα-Fc V1, or SIRPα-Fc V2 (each 5 μg/mL). A) Representative flow cytometry plots showing CFSE dilution (proliferation) of CD4⁺ effector T cells. B) Dot plots show the stimulation indices of CD4⁺ T cells from six paired samples across three independent experiments. Statistical analyses were performed using the Wilcoxon matched-pairs signed rank test. *P* < 0.05 was considered significant. SIRPα, signal-regulatory protein alpha; IgG1, immunoglobulin G1; CFSE, carboxyfluorescein diacetate succinimidyl ester.

To test synergy with other co-stimulatory signals, we performed assays with plate-bound anti-CD28 together with anti-CD3. SIRPα-Fc V2 significantly enhanced CD4⁺ T cell proliferation relative to SIRPα-Fc V1 even with CD28 co-stimulation (median SI, V1 5.02 vs. V2 8.26; *P* = 0.03; Fig. 4).

### Polymorphism of the SIRPα IgV domain modulated the incidence of acute rejection after allogeneic liver transplantation

To investigate clinical relevance, we presumed V9 and NVs as V1-like alleles based on their similarity in SNP repertoires, and we categorized the SIRPα genotype into three groups (V1/V1, V1/V2, and V2/V2). We defined the potential alloimmune response model mediated by recipient and donor APC presentation considering the mechanistic contribution of APC activation through bidirectional SIRPα–CD47 interaction and T cell activation.

**Fig. 5.**
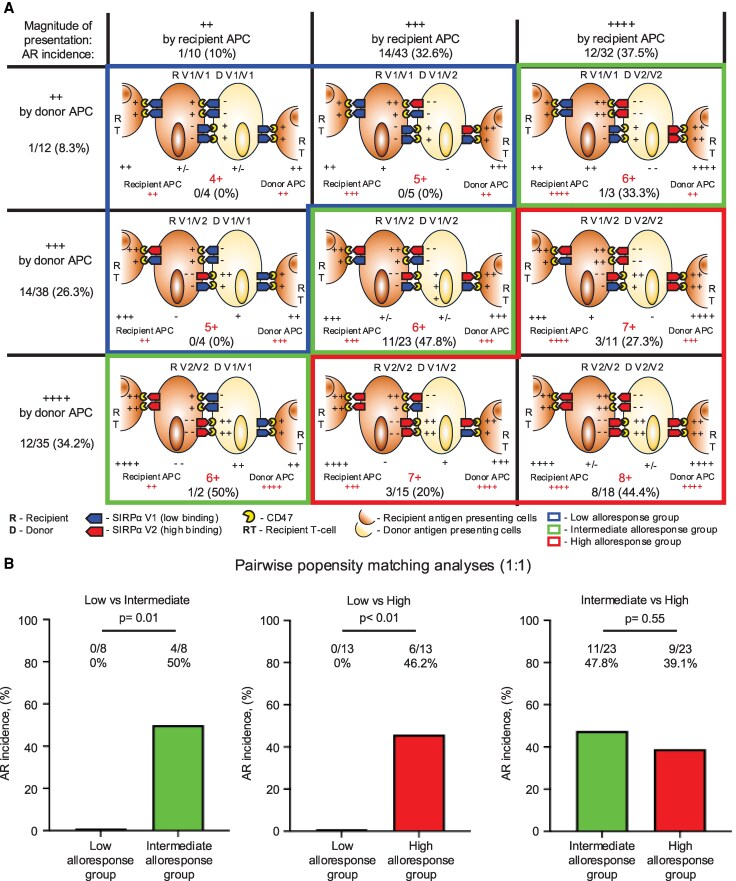
The effect of recipient and donor SIRPα genotype on the incidence of acute rejection after liver transplantation by recipient and donor APC presentation. A) The schemes show that the magnitude of potential alloimmune response model by recipient and donor APC presentation (red “+”) was calculated as the sum of APC and T cell activation (+/−) by SIRPα-CD47 interaction according to recipient and donor SIRPα genotype. We classified into three groups based on the potential magnitude of alloimmune response, 4+ and 5+ as low alloresponse, 6+ as intermediate alloresponse, 7+, and 8+ as high alloresponse group. B) One-to-one propensity score–matched pairwise analyses of acute rejection were conducted between the low and intermediate (left; *n* = 8), low and high (middle; *n* = 13), and intermediate and high (right; *n* = 23) alloresponse groups. SIRPα genotype defined that low alloresponse group showed lower incident of acute rejection after liver transplantation in propensity matched cohort. *P*-value <0.05 was considered as significant.

We semiquantified the signal strength to T cells mediated by CD47-V2 SIRPα binding as ++ and that mediated by CD47-V1 SIRPα binding as +, assuming the sum of the signal strengths determined by the SIRPα alleles as the presumed magnitude of the T cell alloresponse (Fig. 5A). Taking both direct and indirect presentation into account, the potential T cell alloresponse, defined by the SIRPα genotype, was calculated as the sum of the signal strength induced by CD47–SIRPα binding from either the recipient or donor APCs and T cells, resulting in a range from 4+ to 8+. Based on their potential magnitudes, we classified the cases into three groups: 4+ and 5+ as the low alloresponse group, 6+ as the intermediate alloresponse group, and 7+ and 8+ as the high alloresponse group. Among 85 liver transplant recipients managed with double- or triple-drug immunosuppressive regimens including methylprednisolone (MP) and calcineurin inhibitor (CNI), or mycophenolate mofetil (detail in Methods), we observed that the incidence of clinical acute rejection (AR) was increased along with the strength of potential T cell alloresponse defined by the SIRPα genotype. Although alloreactivity measured by CFSE-MLR generally converged to low levels by 3 months post-transplant, persistently high antidonor responses were detected in CD4⁺ T cells specifically within the high-alloresponse group, consistent with our in vitro observations (Fig. S9). To evaluate the statistical significance of T cell signal strength in relation to the SIRPα genotype, we performed one-to-one pairwise propensity score-matched analyses using eight variables: recipient age and sex, primary disease, donor relationship, number of HLA class I mismatches, number of HLA class II mismatches, donor age, and sex to minimize baseline characteristics bias (Supplementary Tables S4–S7). These analyses revealed that the low alloresponse group defined by SIRPα genotype was protective against acute rejection compared with the intermediate- and high-alloresponse groups (Fig. 5B). SIRPα-defined alloresponse groups did not influence long-term clinical outcomes, including incidence of biliary complications, chronic rejection, and overall survival (Fig. S10).

## Discussion

This study explored the impact of SIRPα polymorphisms on T cell activation and the incidence of AR following allogeneic LT. Our overall AR rate (31.8%) falls within the upper range reported in prior studies, which vary considerably in their definitions and monitoring strategies. The use of CFSE-MLR-based immune monitoring in our program likely increased detection compared with biopsy-only definitions (13–16). Consistent with contemporary experience, most episodes were clinically manageable; the principal finding of this study is the association between SIRPα genotype-defined alloresponse and alloimmune events. We focused on both the alloimmune response mediated by APCs and the costimulatory effect of the SIRPα–CD47 interaction on T cells. Our findings highlight the significant role of SIRPα polymorphisms, particularly the V1 and V2 variants, in modulating immune responses through both direct and indirect pathways, with implications for transplant rejection.

Priming T cells with APCs is a critical step that bridges innate and acquired immunity to elicit an alloimmune response (17). In allogeneic transplantation, antigen presentation occurs via both donor and recipient APCs, known as direct and indirect presentation, respectively (18–20). The long-standing passenger leukocyte theory posits that direct presentation is the primary driver of transplant rejection (21). However, subsequent research has convincingly shown that indirect presentation can promote allograft rejection, even immediately after transplantation (22–24). Emerging concepts, such as semidirect presentation, in which donor-derived major histocompatibility complex-peptide complexes are presented by recipient APCs via extracellular vesicle transfer, emphasize that both donor and recipient APCs contribute significantly to the alloimmune response (25–28). Previous studies have demonstrated that a mismatch in SIRPα between donor and recipient triggered innate immune responses through monocyte infiltration and proliferation in an allogeneic bone marrow transplantation model (6). Garcia-Sanchez et al. (29) investigated the impact of SIRPα genotype on innate allorecognition via the SIRPα–CD47 axis in HLA-identical kidney transplant recipients and reported a significantly higher incidence of histological peritubular capillaritis, without a corresponding increase in rejection, in SIRPα-mismatched pairs. Along similar lines, Zhao et al. (30) recently demonstrated the clinical significance of SIRPα–CD47 mismatch in kidney transplant cohorts included both HLA-identical and -mismatched pairs, with the vast majority receiving T-cell-depleting induction (>97%), thereby establishing this axis as an innate myeloid determinant of allograft outcomes (acute rejection and long-term graft survival). The discrepancy between their findings and ours may reflect differences in organ type and in induction immunosuppression strategies. Modulation of APC function by SIRPα IgV domain polymorphisms likely influences alloimmune responses through both donor and recipient APCs in allogeneic solid organ transplantation. Herein, our study extends these observations by providing evidence that SIRPα polymorphisms not only modulate monocyte CD47 binding but also directly potentiate CD4⁺ T-cell costimulation. Several studies have demonstrated that agonistic anti-CD47 antibodies can activate T cells by providing costimulatory signals (9–11). Additionally, SIRPα ligation of CD47 has been linked to protein kinase theta signaling, which is involved in T cell activation (31). In xenogeneic hematopoietic transplantation models, CD47 signaling has been shown to regulate T cell homeostasis, as demonstrated by functional SIRPα–CD47 signaling in mice and SIRPα knockout models (32, 33). In cancer immunology, evidence suggests that SIRPα–CD47 interactions enhance cell-cell adhesion and T cell cytotoxicity against tumors (34). Despite these findings, the costimulatory role of SIRPα–CD47 in T cells has not garnered much attention in the era of immune checkpoint inhibitors. Our data show that the V2 SIRPα variant significantly enhanced the proliferation of CD4^+^ T cells under polyclonal stimulation, in synergy with CD28 co-stimulation. These findings support a model in which SIRPα polymorphisms preferentially potentiate CD4⁺ T cell costimulation and helper functions—such as dendritic-cell licensing and priming of alloreactive CD8⁺ T cells (31–33)—thereby promoting a CD8-mediated rejection phenotype despite minimal direct effects on CD8⁺ proliferation in vitro (35–37). SIRPγ is encoded within the same genomic cluster as SIRPα, is expressed on human (but not murine) T cells, where it modulates T cell responses through CD47 via its IgV binding domain (38). We did not genotype SIRPG or quantify SIRPγ expression, and, to our knowledge, polymorphisms within the SIRPγ IgV domain have not been investigated. Because we did not assess linkage disequilibrium between SIRPA and SIRPG, a contribution of SIRPγ to the observed clinical associations cannot be excluded. Our findings should therefore be interpreted with this limitation in mind and provide the need for future studies directly examining SIRPG variation and SIRPγ expression in transplantation. The biological effects of SIRPα polymorphisms can be explained by their differential binding capacities. Previous studies have shown that the binding capacity of human SIRPα variants to CD47 varies significantly (5, 6). Rodriguez et al. first demonstrated that V2 SIRPα has a stronger binding capacity than V1, a finding that has been reproduced by other groups in various contexts (39–41). However, the significance of these differences may depend on the characteristics of the experimental system used (i.e. type of immortalized cell line and culture conditions), as some studies have found statistical significance while others did not (40, 41). In this study, we demonstrated for the first time that V2 SIRPα exhibited higher binding capacity to CD47 on freshly isolated human monocytes, which correlated with a stronger costimulatory effect on CD4^+^ T cells.

The SIRPα IgV domain is highly polymorphic, with at least 10 variants identified in humans (5). We observed a unique distribution in the Japanese cohort, with V1 (35.7%) and V2 (62.3%) as the dominant variants. The predominance of V2 in this population is consistent with the findings that SIRPα V2 is more common in East Asian populations (42). This relatively low genetic diversity may reflect the unique demographic characteristics of Japan. Our comprehensive genotyping using both Sanger sequencing and NGS revealed new variants and highlighted the potential to apply SNP-based recognition of SIRPα polymorphisms in diverse populations globally.

This study had several limitations. First, the sample size was relatively small, particularly in the low-alloresponse group, which may limit the generalizability of our findings. Additionally, the complexity of the SIRPα–CD47 signaling axis, involving both innate and adaptive immune responses, warrants further investigation to fully elucidate the mechanisms underlying its role in transplantation. Future studies with larger cohorts and more diverse patient populations are needed to validate our findings and explore the potential of SIRPα genotyping as a predictive tool in clinical practice.

In conclusion, this study demonstrates that SIRPα polymorphisms are pivotal in modulating immune responses through both APCs and T cells in allogeneic LT. The differential binding capacity of SIRPα variants, particularly V2, to CD47 significantly affects the costimulatory signals in CD4^+^ T cells. Moreover, we generalize these effects to liver transplantation and present an NGS-based, population-aware genotyping framework together with a clinically actionable, genotype-derived alloresponse score for acute rejection risk. These findings suggest that SIRPα genotyping could serve as one component of multiparameter risk models, advancing precision medicine and transplant immunobiology.

## Methods

### Study subjects

The in vitro study to analyze the impact of the SIRPα genotype on binding capacity, T cell proliferation, and APC phenotype used PBMCs obtained from 36 healthy volunteers. Samples for binding and phenotype assay were selected based on the SIPRα genotype of either homogenous V1/V1 or V2/V2 from the healthy volunteer cohort (*n* = 94) using the Sanger sequence method.

In this cohort study, 154 recipients who received primary living donor LT at Hiroshima University Hospital from October 2006 to February 2021 and their corresponding donors were enrolled. In the analysis of clinical outcomes, 44 recipients received adoptive immunotherapy with activated liver graft-derived lymphocytes to prevent the recurrence of hepatocellular carcinoma (43), and 25 antidonor antibody-positive recipients received desensitization therapy. These cases were excluded from the analysis owing to concerns about immunological modification. The remaining 85 patients were managed with dual- or triple-drug immunosuppression comprising MP and a CNI, with CNI-sparing regimens incorporating mycophenolate mofetil (MMF) for patients with chronic kidney disease (individual data in Table S8). Tacrolimus was the standard CNI; however, cyclosporin A was used in eight patients with HCV infection because of its reported suppression of HCV replication in the predirect-acting antiviral era (44). In the absence of an immunological events, methylprednisolone was tapered over the course of 1 year. Immune monitoring by mixed lymphocyte reaction (MLR) was performed at pre-LDLT and 1, 2, 3, and 4 weeks, as well as at 3 months post-LDLT, contingent on the availability of donor material. Briefly, a CFSE-based flow cytometric MLR (CFSE-MLR) was used to quantitatively monitor antidonor alloresponses in CD4^+^ and CD8^+^ T cells. One-way MLRs assessed responses to self, donor, and ABO blood type-matched third-party stimulators. After 5 days of incubation, the proliferation index (PI) was calculated from CFSE-dilution (division) profiles of reactive T cells, and the stimulation index (SI) was defined as the ratio of allogeneic PI to self-response PI (16). Use of CNI and MMF was comparable across the three alloresponse groups, irrespective of acute rejection (AR) occurrence (Table S9 and Fig. S11). Clinical rejection was suspected based on elevations in hepatic enzymes (transaminases, γ-glutamyltransferase) and/or bilirubin. After a diagnostic evaluation to exclude alternative causes of graft dysfunction (e.g. infections, vascular or biliary complications, or recurrence of the primary disease), rejection was diagnosed by CFSE-MLR and/or biopsy. CFSE-MLR, originally developed at our center as a noninvasive immune-monitoring method, has been validated for the sensitive and specific detection of antidonor responses (16); accordingly, we preferentially use CFSE-MLR to minimize biopsy-related risks. Episodes of AR were initially treated with steroid pulse (125–500 mg intravenous MP for 3 days or more), according to the clinical severity of AR, with a gradual tapering of the dose and return to the previous oral double-drug regimen. Rejection was considered steroid-resistant AR when liver function tests improved by <50% of the highest values after three steroid boluses. Episode of clinical AR occurring within 90 days post-transplant—defined as those treated with steroid pulse as first-line therapy—were recorded. For steroid-resistant AR, antithymoglobulin was administered in 12 of 27 patients. Among the 27 AR episodes, 17 were diagnosed by CFSE-MLR (including 3 also confirmed by biopsy), 5 by biopsy alone (including the same 3 also positive by CFSE-MLR), and the remaining 8 were treated based on clinician judgment. Banff Rejection Activity Index (RAI) scores for biopsy-proven cases are shown in Table S10. The study was conducted in accordance with the principles of the Declaration of Helsinki and its amendments. The study protocol was approved by the Institutional Review Board of Hiroshima University (Hi-77 and C-290), and written informed consent was obtained from all participants.

### Genomic DNA extraction

The genomic DNA (gDNA) was extracted from PBMCs using the Wizard SV Genomic DNA Purification System (Promega Corporation, Madison, WI) or from whole blood using QIA cube equipment (QIAGEN) with the QIAmp DNA Mini kit (QIAGEN) according to the manufacturer's protocol. The obtained gDNA concentration was quantified and quality checked by the Nanodrop 2000c Spectrophotometer (Thermo Fisher Scientific) and stored at 4 °C until use.

### SIRPα genotyping using the sanger sequence method

First, 720 bp sequence alignment of the SIRPα IgV domain (exon 3 of SIRPα) was amplified with the primers described by Takenaka et al. (5). Sanger sequencing was performed as previously described (45). Briefly, polymerase chain reaction (PCR) was performed using the Quick Taq HS Dye Mix (TOYOBO). The thermal cycling (Bio-Rad S1000) condition was initial denaturation at 94 °C for 5 min, followed by 40 cycles of 94 °C for 30 s, annealing 60 °C for 30 s, extension at 72 °C for 30 s, and final extension at 72 °C for 7 min. The 720 bp size of the expected amplicons was detected and excised through electrophoresis. Target amplicons were purified using the QI Quick Gel Extraction Kit (QIAGEN) according to the manufacturer's protocol. Purified PCR products were labeled with the Big Dye Terminator v3.1 sequencing kit (Applied Biosystems) and were directly sequenced in an ABI PRISM 3130xl genetic analyzer (Applied Biosystems). Seascapes Software v2.7 (Applied Biosystems) was used to analyze, assemble, and generate consensus nucleotide sequences.

### SIRPα genotyping by next-generation sequencing

NGS analysis was performed using gDNA. Genomic libraries were prepared using 15 ng of the gDNA template and sequenced using Illumina's standardized protocol (Nextera XT DNA Library kit). Briefly, a 720 bp sequence alignment was amplified using the primers described by Takenaka et al. The amplicons were purified and tormented. The index primers were attached to each sample in 12 PCR cycles. The PCR products were purified using the magnetic bead purification method. The final amplicon pool of the samples was loaded onto a Misses System (v2 Chemistry). Sequencing was performed on both ends for 250 cycles.

The sequences obtained were processed using a free online Galaxy analysis pipeline (https://usegalaxy.org). FASTQ files were uploaded, low-quality scores (average < 20), and adapters were trimmed using the Trimmomatic tool (46) for paired-end sequences.

High-quality sequencing data were generated by removing low-quality, duplicate, and shorter reads (<35 bp). A minimum quality score of Q30 (corresponding to a 1:1,000 error rate) was required for a minimum of 90% of the sequenced bases, and runs that failed these metrics were excluded. The cluster density in each run was 600/800, indicating a unique depth of coverage across the capture region of the 3,000 amplicons. Medium and long reads (>100 bp) were aligned to the reference genome (GRCh37/hg19). Variant calls were made using the FreeBayes Bayesian Genetic variant detector (47). We considered data showing the following parameters acceptable for analyses: sequencing coverage > 100× (bi-directional true paired-end sequencing), quality of 100, and a variant frequency ≥ 3%.

Discrepancies between samples and reference genomes were detected, and their types, such as deletions, insertions, and SNPs, were determined. To annotate the sequence variants, Integrative Genomics Viewer software (UC San Diego, Broad Institute of MIT) was used. We deposited these files into the NCBI Sequence Read Archive Bioproject number: PRJNA1345187.

### Flow cytometry

The following monoclonal Abs (mAbs) were used for flow cytometric analyses: PE-CD172a (15–414), APC-Cy7-CD11b (ICRF44), PE-Cy7-CD86 (IT2.2), APC-Cy7-human leukocyte antigen (HLA)-DR (L243), PE-Cy7-CD8a (HIT8a), PE-CD4 (OKT4), PE-Dazzle 594-CD45RA (HI100), BV711-CCR7 (G043H7), and APC-CD25 (M-A251), all were purchased from Biolegend (San Diego, CA, USA). APC-CD14 (M5E2) was purchased from BD Biosciences (San Jose, CA, USA). Dead cells were excluded from analysis using the forward Zombie Aqua Fixable Viability Kit (BioLegend), or 7-aminoactinomycin D (7-AAD; BD Biosciences) staining. Data were collected on a FACS Canto II (BD Biosciences, Mountain View, CA, USA) and analyzed using FlowJo version 10 (Tree Star, Ashland, OR, USA).

### SIRPα binding assay

The binding capacity of SIRPα to CD47 was assessed using rhCD47-Fc protein with PBMCs that were collected from volunteers to determine their SIRPα genotype (V1/V1 or V2/V2). The PBMCs were isolated through density gradient centrifugation of heparinized blood using Lympholyte (Cedarlane Laboratories, Ontario, Canada). PBMCs were either used freshly or after activation with 5 µg/mL LPS for 3 days, following the manufacturer's protocol. Before the assay, PBMCs were labeled with PE-SIRPα, APCCD14, and APC-Cy7-CD11b mAb that recognize SIRPα expressing cells. Recombinant CD47-Fc protein (R&D, USA) was labeled with the Alexa Fluor 488 antibody labeling kit (Invitrogen). Prestained PBMCs were incubated with recombinant CD47-Fc protein at serially diluted concentrations ranging between 25 and 2,500 nM for 1 h at 4°C. The binding capacity was measured as the mean fluorescence intensity (MFI) of Alexa Fluor 488 gated on CD14^+^ CD11b^+^ cells.

### T cell proliferation assay

To investigate the differential impact of SIRPα protein V1 and V2 genotype (Supplementary material information) as ligands, T cell proliferation was assessed under CD3 stimulation. CD8^+^ and CD4^+^CD25^−^ T cells were isolated by FACS sorting on FACSAria Fusion Flow Cytometer (BD), following pan T cell isolation via negative selection using the Human Pan T Cell Isolation Kit (Miltenyi Biotec, Bergisch Gladbach, Germany) in an autoMACS Pro Separator (Miltenyi Biotec), according to the manufacturer's instructions. Additional memory-phenotype staining with CD45RA and CCR7 was performed for isolate of CD45RA⁺CCR7⁺ naïve and effector-memory subpopulations (CD45RA⁺CCR7⁻, CD45RA⁻CCR7⁺, and CD45RA⁻CCR7⁻) (Fig. S8). 96-well flat-bottom plates (Falcon) were coated by anti-CD3 Ab (5 µg/mL) with either human SIRPα-Fc recombinant protein V1 genotype (BioLegend), V2 genotype (LSBio), or control human IgG1 (R&D) at a final concentration of 5 µg/mL in sterile phosphate-buffered saline incubated 2 h at 37°C. To investigate co-stimulatory synergy, experiments were performed using plates pre-coated with anti-CD3 Ab (5 µg/mL) and anti-CD28 antibody (10 µg/mL). CFSE-labeled isolated T cells were cultured at 1 × 10^6^/mL in 200 µL of complete RPMI medium (RPMI 1640 medium [Nacalai Tesque, Kyoto, Japan] supplemented with 5% fetal bovine serum [SERANA, Pessin, Germany], 100 mM sodium pyruvate [Thermo Fisher Scientific, Waltham, MA], 100 U/mL penicillin–streptomycin [Thermo Fisher Scientific], 1% HEPES buffer [Thermo Fisher Scientific], and 50 µM 2-ME) in the prepared microwells for 5 days. All assays were performed in triplicates. Cells were stained with anti-CD4 or anti-CD8 mAbs and analyzed using a FACSCanto II (BD Biosciences, Mountain View, CA, USA). For qualificative estimation, divisions of reactive T cells, identiﬁed using their Carboxyfluorescein succinimidyl ester (CFSE) intensities, labeled from 0 to *n*, were the dividing time. A single cell that divides n times generates 2*^n^* daughter cells. Using this mathematical relationship, the absolute numbers of precursors and proliferation events were extrapolated from the number of daughter cells in each division. Proliferation indices (PIs) in the CD4^+^ and CD8^+^ T cell subsets were calculated as the ratio of proliferation events per precursor. The stimulation indices were calculated as the normalized PI based on the PI of the IgG control.

### Statistical analysis

Nonparametric Mann–Whitney, Wilcoxon matched-pairs and chi-square tests were performed to compare the two variables. For multiple group comparisons, we used a one-way analysis of variance followed by Tukey's post hoc test. To adjust for baseline differences across the three alloresponse groups (low, intermediate, and high), 1:1 pairwise propensity score–matching analyses were performed using each patient's estimated propensity score. Covariates included recipient age; recipient sex (male, female); primary disease (autoimmune vs. other); donor relationship (first-degree relative vs other); number of HLA class I mismatches; number of HLA class II mismatches; donor age; and donor sex (male, female). Propensity-score models were constructed for each group comparison, and 1:1 nearest-neighbor matching with a caliper of 0.1 yielded 8, 13, and 23 matched pairs for the low vs. intermediate, low vs high, and intermediate vs high comparisons, respectively. Statistical analyses were performed using Prism 10 (GraphPad Software, San Diego, CA, USA) and JMP Pro 17 software (SAS Institute). Differences were considered significant if the *P*-value was <0.05.

## Supplementary Material

pgaf351_Supplementary_Data

## Data Availability

The next-generation sequencing data generated in this study is available in the NCBI Sequence Read Archive under BioProject accession number PRJNA1345187. All other data are included in the article and/or Supplementary materials.
