# Supplementary material for: Impact of SIRPα genotype combinations in recipients and donors on alloimmune response in liver transplantation
Source: PNAS Nexus. 2025 Nov 5;4(11):pgaf351. doi: 10.1093/pnasnexus/pgaf351 (PMC12617414; doi:10.1093/pnasnexus/pgaf351)
Supplement: pgaf351_Supplementary_Data [file pgaf351_supplementary_data.pdf]

Supplementary Information for
"Impact of SIRPα Genotype Combinations in Recipients and Donors on Alloimmune Response in Liver Transplantation"

Akhmet Seidakhmetov, Naoki Tanimine, Yuka Tanaka, Ryosuke Arata, Ryosuke Nakano, Hiroshi Sakai, Masahiro Ohira, Hiroyuki Tahara, Kentaro Ide, Tsuyoshi Kobayashi and Hideki Ohdan

Corresponding author Hideki Ohdan.
Email: hohdan@hiroshima-u.ac.jp

This PDF file includes:
Figures S1 to S11
Tables S1 to S10
Supplementary material information

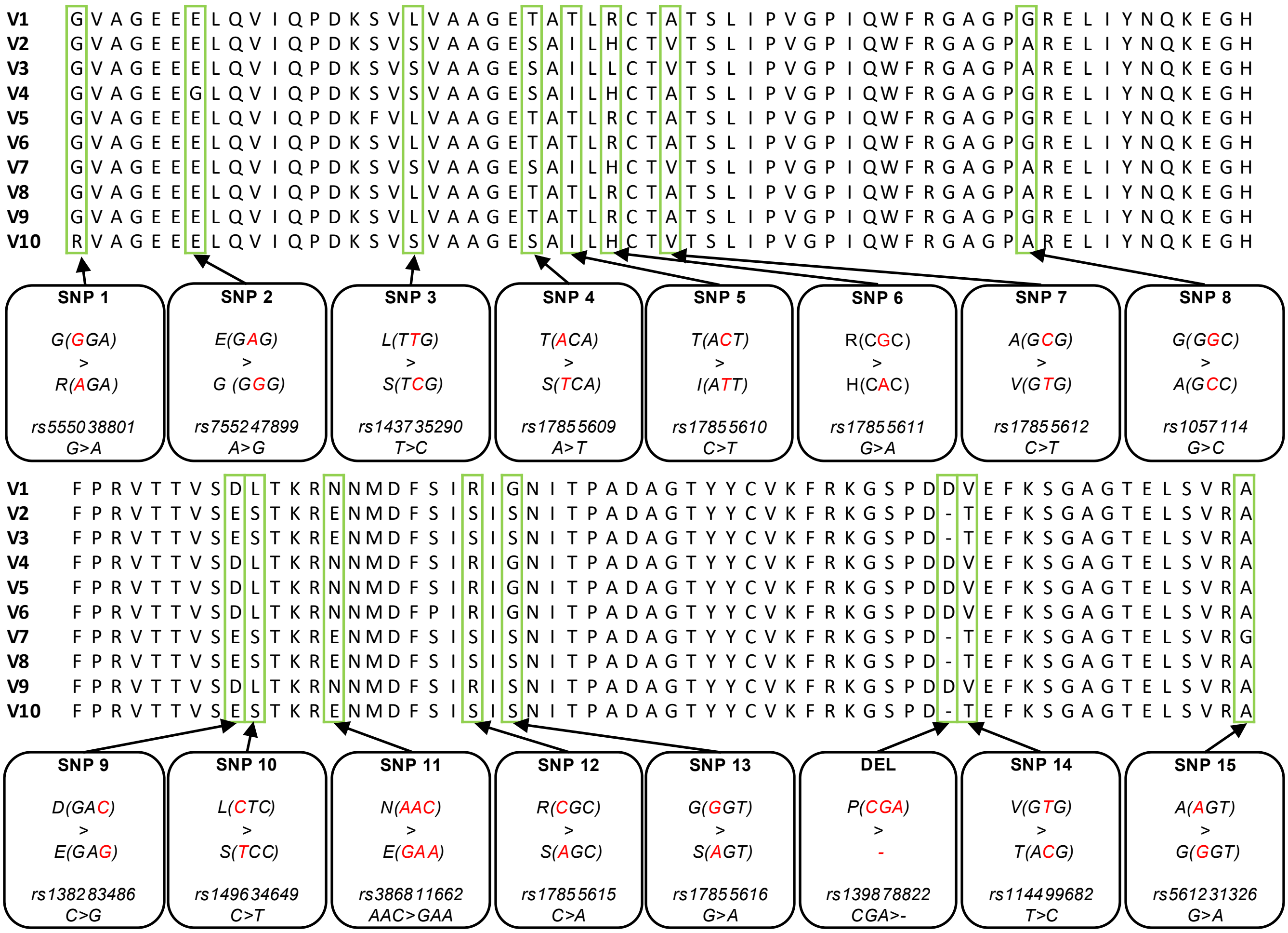

Supplementary Figure 1. Sequence alignment of the human SIRPα amino acid variants

The sequences are from the previous publication by Takenaka et al. [5]. The residues that differ between the different variants are highlighted in green squares. The variants can be converted to the SNP repertoires.

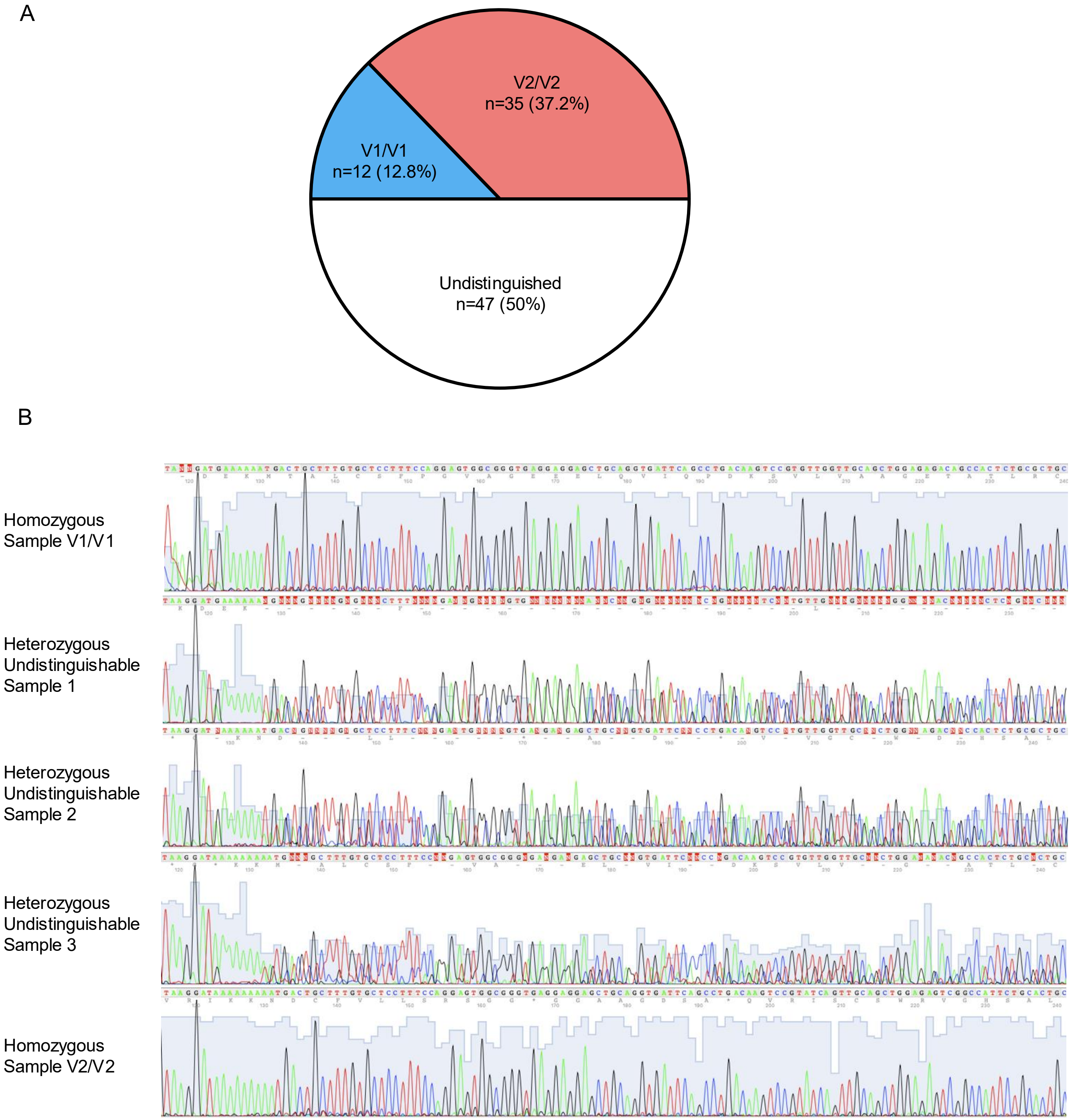

Supplementary Figure 2. The SIRPα genotypes defined by the Sanger sequencing distribution in the healthy volunteer cohort and representative results of the homozygous and heterozygous volunteer samples

(A). Genotype distribution of the homozygous and heterozygous volunteer samples (B). The homozygous samples showed clear peaks, however the heterozygous samples were not distinguishable peaks in chromatogram.

	SNP 1 G(GGA) > R(AGA) rs555038801 G>A	SNP 2 E(GAG) > G (GGG) rs755247899 A>G	SNP 3 L(TTG) > S(TCG) rs143735290 T>C	SNP 4 T(ACA) > S(TCA) rs17855609 A>T	SNP 5 T(ACT) > I(ATT) rs17855610 C>T	SNP 6 R(CGC) > H(CAC) rs17855611 G>A	SNP 7 A(GCG) > V(GTG) rs17855612 C>T	SNP 8 G(GGC) > A(GCC) rs1057114 G>C
**V1**	G	A	T	A	C	G	C	G
**V9**	G	A	T	A	C	G	C	G
**NV1**	G	A	T	A	C	G	C	C
**NV2**	G	A	T	A	C	G	C	G
**V2**	G	A	C	T	T	A	T	C

	SNP 9 D(GAC) > E(GAG) rs138283486 C>G	SNP 10 L(CTC) > S(TCC) rs149634649 C>T	SNP 11 N(AAC) > E(GAA) rs386811662 AAC>GAA	SNP 12 R(CGC) > S(AGC) rs17855615 C>A	SNP 13 G(GGT) > S(AGT) rs17855616 G>A	DEL P(CGA) > - rs139878822 CGA>-	SNP 14 V(GTG) > T(ACG) rs114499682 T>C	SNP 15 A(AGT) > G(GGT) rs561231326 G>A
**V1**	C	C	A	C	G	CGA	T	G
**V9**	C	C	A	C	A	CGA	T	G
**NV1**	C	C	A	C	G	CGA	T	G
**NV2**	G	T	G	A	A	CGA	T	G
**V2**	G	T	G	A	A	-	C	G

Supplementary Figure 3. The SNP haplotypes of SIRPα genotypes

SIRPα haplotype was consisted of 15 SNPs and 1 CGA deletion. The common variants V1 and V2 showed differential SNPs in 16 locations. The rare variants V9 and new variants NV1 and NV2 showed similarity in the SNP repertories with V1. Abbreviations: SNP, single nucleotide polymorphism; DEL, deletion.

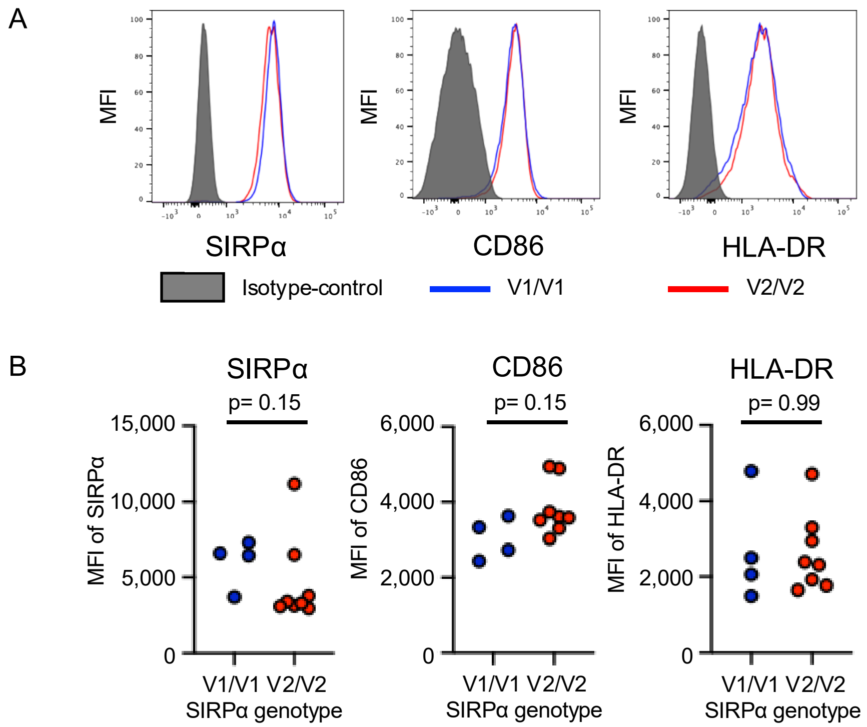

Supplementary Figure 4. Expression of SIRPα and antigen presentation related molecules on monocytes

PBMC from healthy volunteer genotyped as V1/V1 or V2/V2 were examined by flow cytometry. (A) Representative histograms and (B) bar graph show the expression of SIRPα, CD86 and HLA-DR on $CD14^+$ monocytes and mean fluorescence intensity (MFI) (V1/V1 n=4, V2/V2 n = 8).

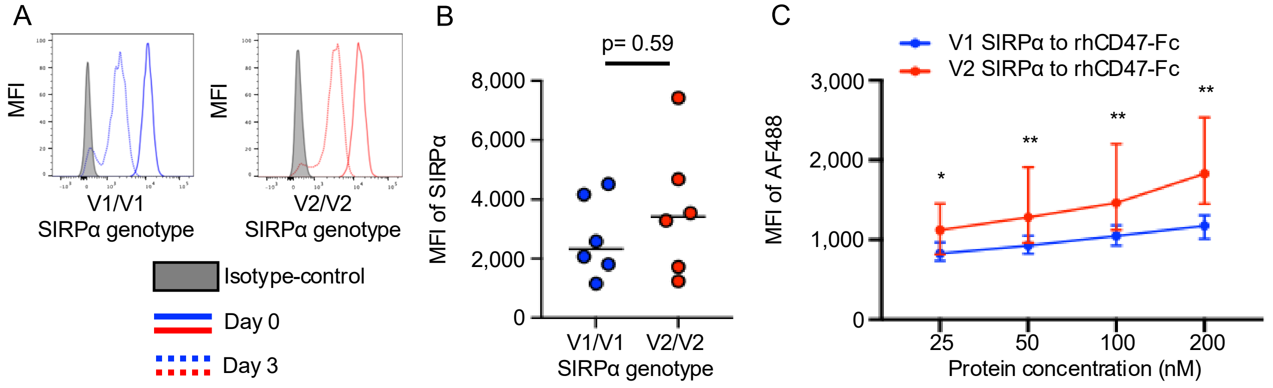

Supplementary Figure 5. The binding capacity of SIRPα V2 was superior to SIRPα V1 even after LPS stimulation.
Peripheral blood mononuclear cells (PBMCs) from healthy volunteers with V1/V1 (blue) and V2/V2 (red) SIRPα genotypes were stimulated with Lipopolysaccharide (LPS) 5 µg/mL for 3 days. Pre-activated PBMCs from healthy volunteers were incubated with Alexa Fluor 488 binding recombinant human CD47-Fc protein. MFI of Alexa Fluor 488 (AF488) was evaluated as binding capacity of SIRPα on monocytes (gated on $CD14^{+}CD11b^{+}$) with CD47 protein. (A) Representative histograms of SIRPα expression on $CD14^{+}CD11b^{+}$ monocytes after 3-day incubation with LPS. (B) bar graph show the SIRPα expression (V1/V1 n = 6, V2/V2 n = 6). (C) V2 SIRPα shows higher binding capacity than V1 (n = 6 from 4 experiments). Median ± range shown; Statistical analyses were performed using Mann-Whitney U test. $p < 0.05$ was considered significant. Abbreviations: SIRPα, signal-regulatory protein alpha; MFI, mean fluorescent intensity; * $p < 0.05$, ** $p < 0.01$

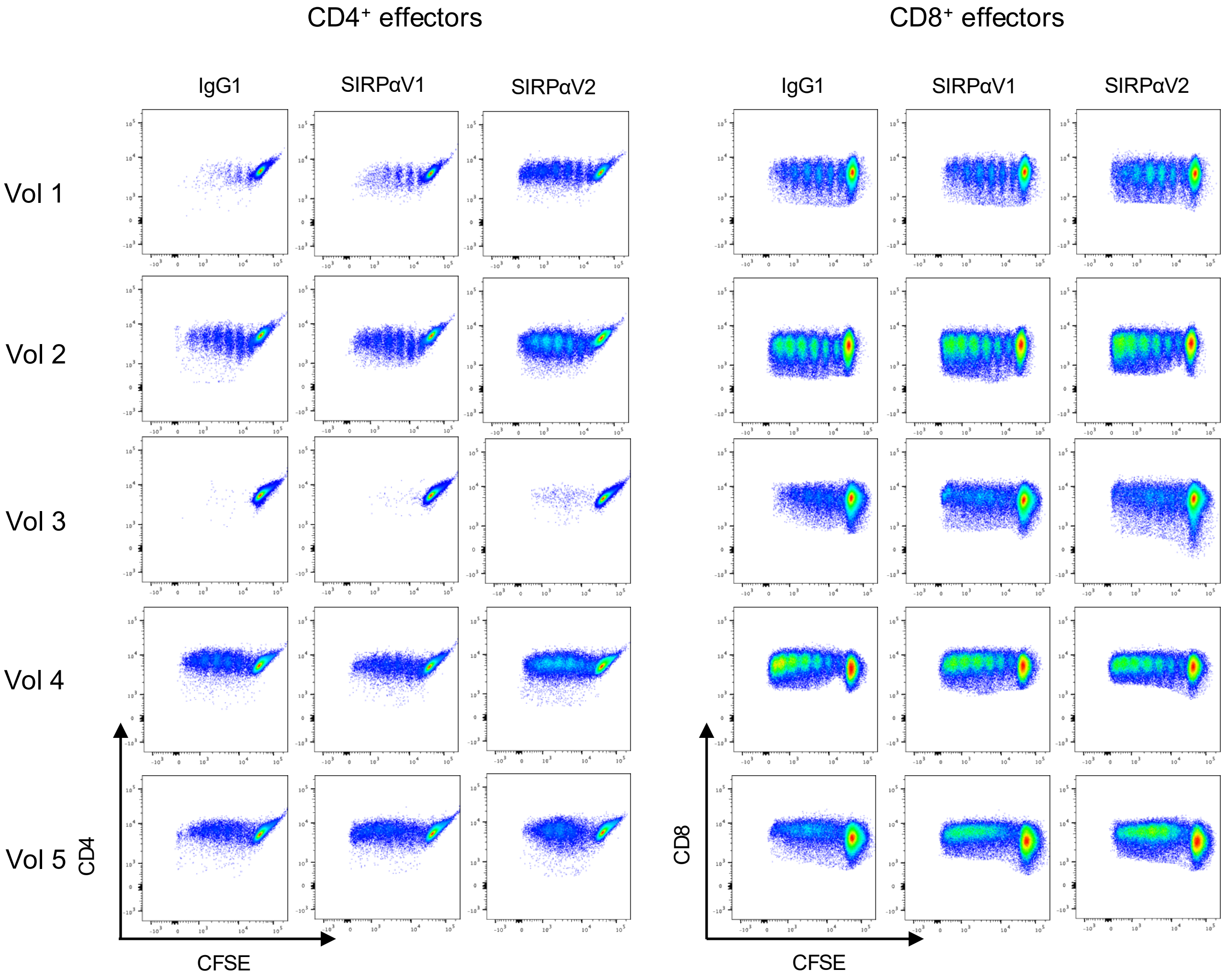

Supplementary Figure 6. Individual flow cytometry plots from the T-cell proliferation assay. Plots show proliferation (CFSE dilution) of effector CD4^{+}T cells (left) and CD8^{+}T cells (right) stimulated with plate-bound anti-CD3 antibody (5 µg/mL) in the presence of an IgG1 isotype control, SIRPα-Fc V1, or SIRPα-Fc V2 (each 5 µg/mL). Abbreviations: SIRPα, signal-regulatory protein alpha; CFSE, carboxyfluorescein diacetate succinimidyl ester

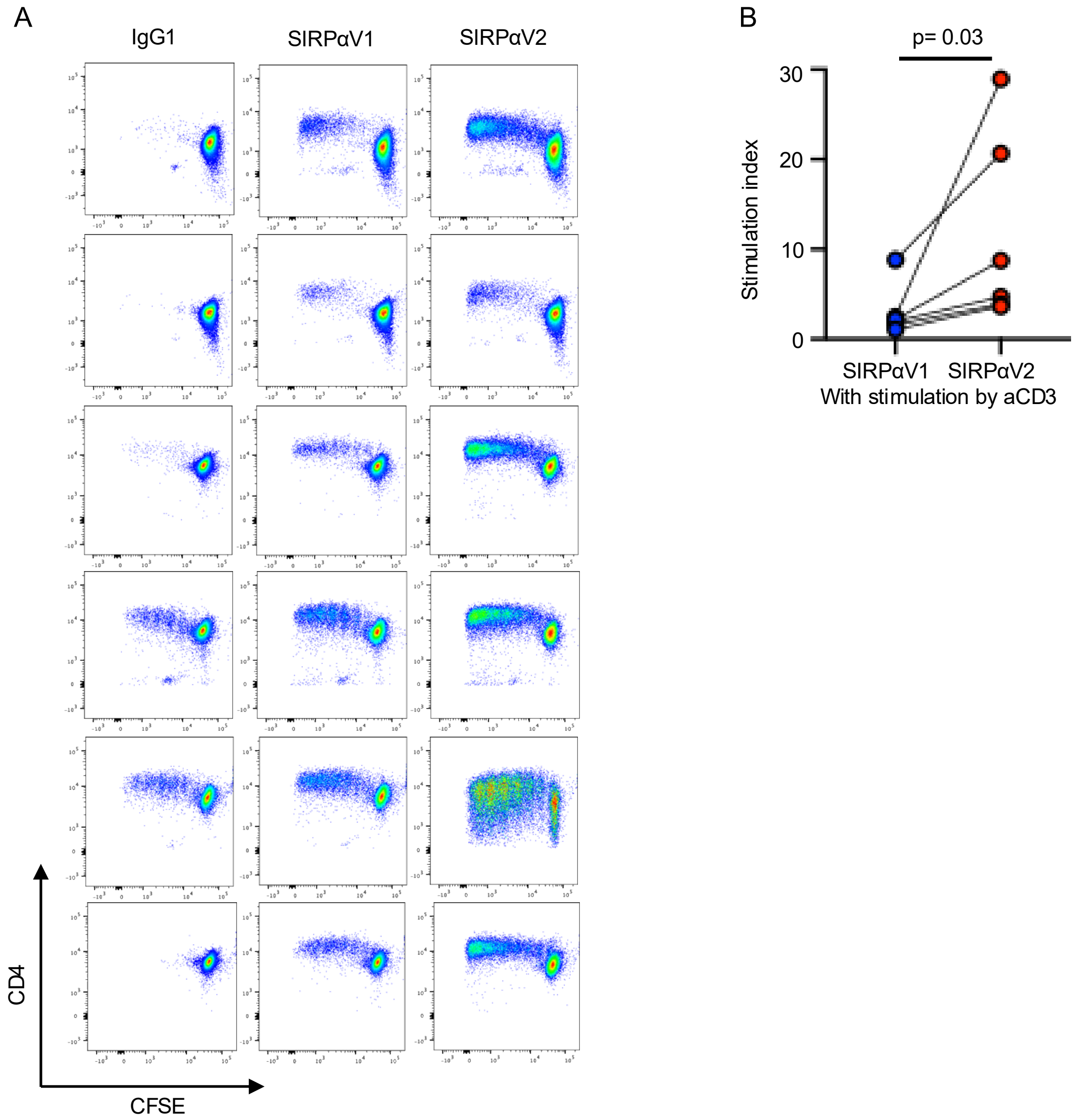

Supplementary Figure 7. The effect of SIRPα-Fc V2 on $CD4^+CD25^-$ T cells is reproducible. CFSE-labeled $CD4^+CD25^-$ T cells were FACS-sorted from healthy-donor PBMCs and cultured for 5 days with plate-bound anti-CD3 (5 µg/mL) in the presence of an IgG1 isotype control, SIRPα-Fc V1, or SIRPα-Fc V2 (each 5 µg/mL). (A) Representative flow cytometry plots showing CFSE dilution (proliferation) of $CD4^+$ effector T cells. (B) Dot plots show stimulation indices for $CD4^+$ T cells from six donors across three independent experiments (N = 3; n = 6 donors); four of the six donors overlap with those in Figure 3. Statistical analyses were performed using the Wilcoxon matched-pairs signed rank test. $p < 0.05$ was considered significant. Abbreviations: SIRPα, signal-regulatory protein alpha; IgG1, immunoglobulin G1; CFSE, carboxyfluorescein diacetate succinimidyl ester

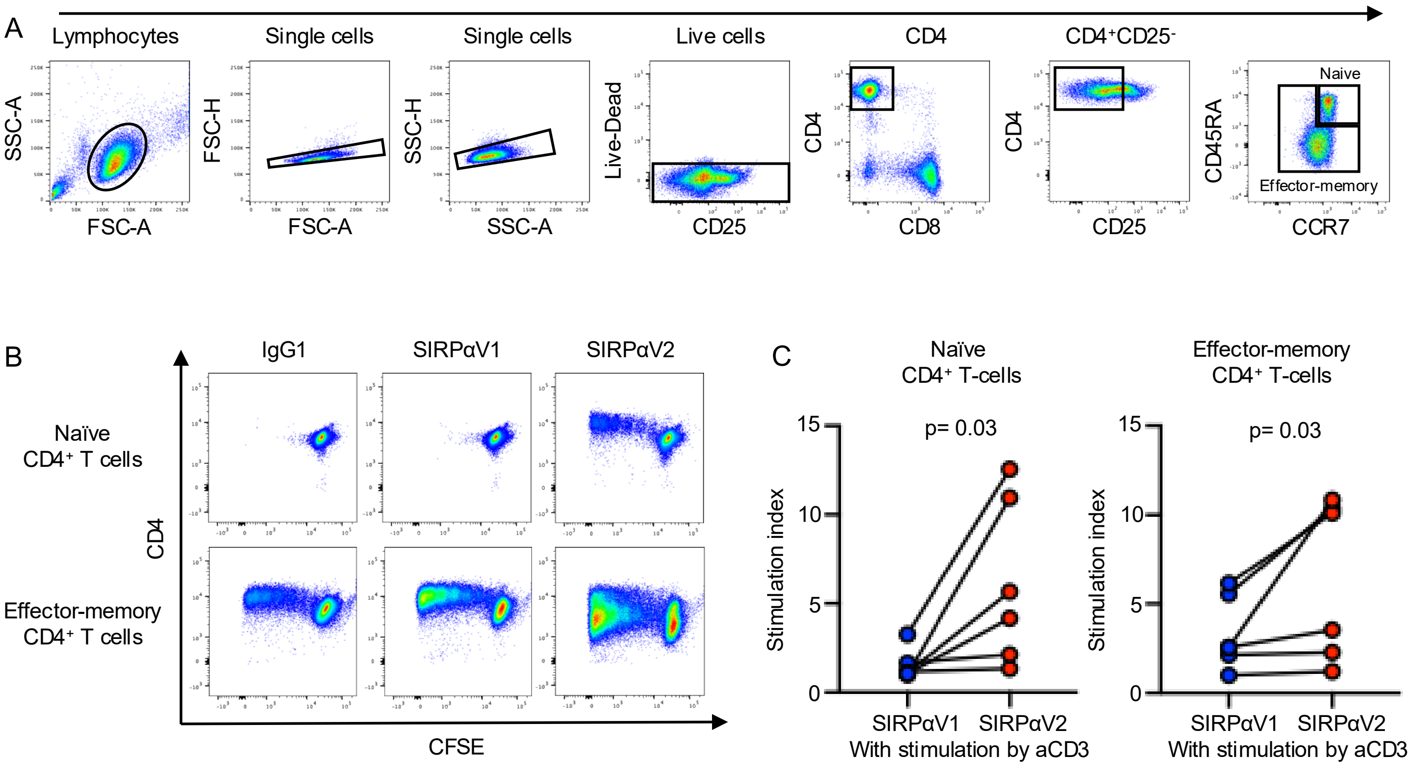

Supplementary Figure 8. SIRPα-Fc V2 protein enhances the proliferation of naïve and effector-memory CD4$^+$ T cells during TCR stimulation relative to SIRPα-Fc V1.

CFSE-labeled CD45RA$^+$CCR7$^+$ naïve and effector-memory (E-M) CD4$^+$ T cells were cultured for 5 days with plate-bound anti-CD3 antibody (5 µg/mL) in the presence of an IgG1 isotype control, SIRPα-Fc V1, or SIRPα-Fc V2 (each 5 µg/mL). (A) Gating strategy for isolating naïve and E-M CD4$^+$ T-cell subsets. (B) Representative flow cytometry plots showing CFSE dilution (proliferation) in naïve and E-M CD4$^+$ T cells. (C) Dot plots show stimulation indices for CD4$^+$ T cells from six donors across three independent experiments (N = 3; n = 6 donors). Statistical analyses were performed using the Wilcoxon matched-pairs signed rank test. $p < 0.05$ was considered significant. Abbreviations: SIRPα, signal-regulatory protein alpha; CFSE, carboxyfluorescein diacetate succinimidyl ester

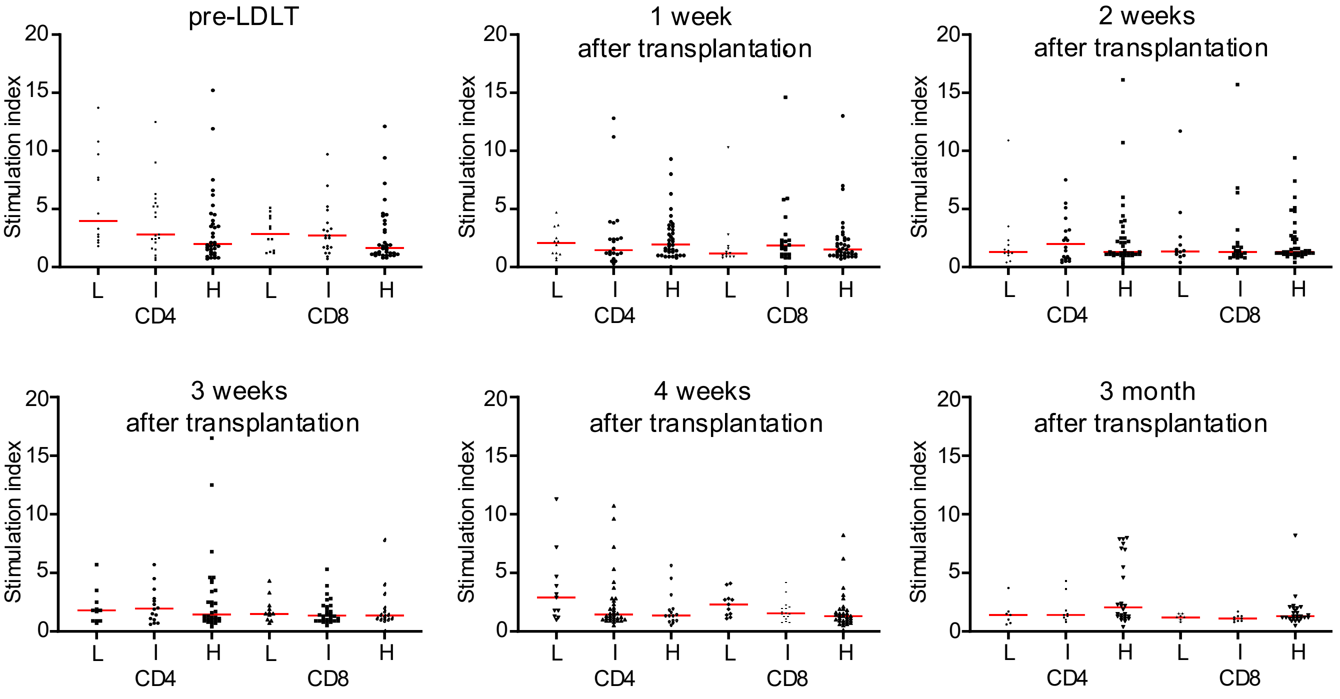

Supplementary Figure 9. Anti-donor CD4^{+}alloresponses detected by immune monitoring after living-donor liver transplantation (LDLT). Mixed lymphocyte reaction (MLR) assays were performed using recipient peripheral blood mononuclear cells (PBMCs) as responders and irradiated donor PBMCs as stimulators. Proliferation was quantified by flow cytometry preoperatively and at weeks 1–4 and at 3 months after LDLT.

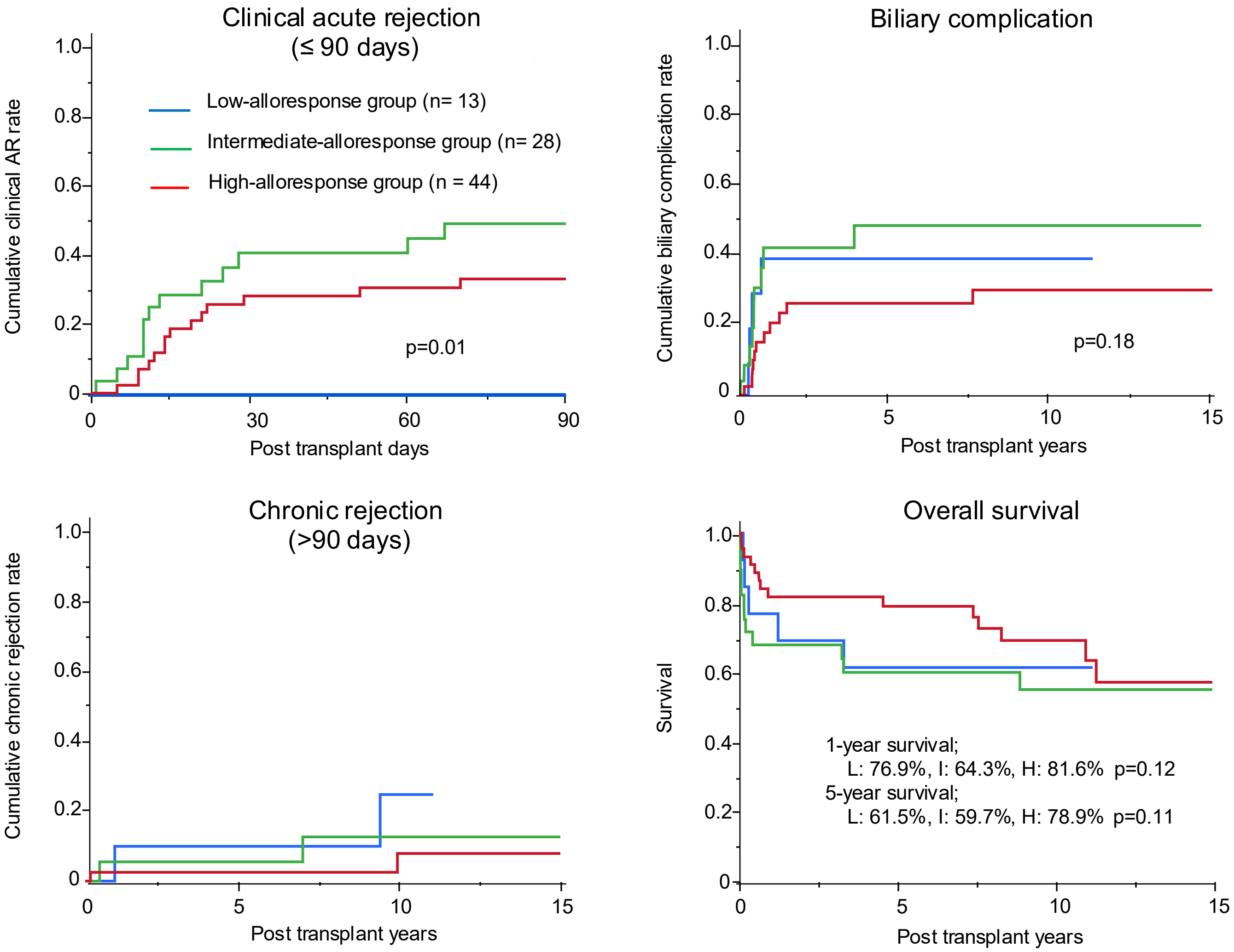

Supplementary Figure 10. Clinical outcomes after living-donor liver transplantation. Kaplan–Meier curves for cumulative clinical acute rejection, biliary complications, chronic rejection, and overall survival were generated for 85 patients and stratified by low, intermediate, and high alloresponse groups defined by recipient–donor SIRPα genotype. Group comparisons were assessed using the Wilcoxon signed-rank test; $p < 0.05$ was considered statistically significant.

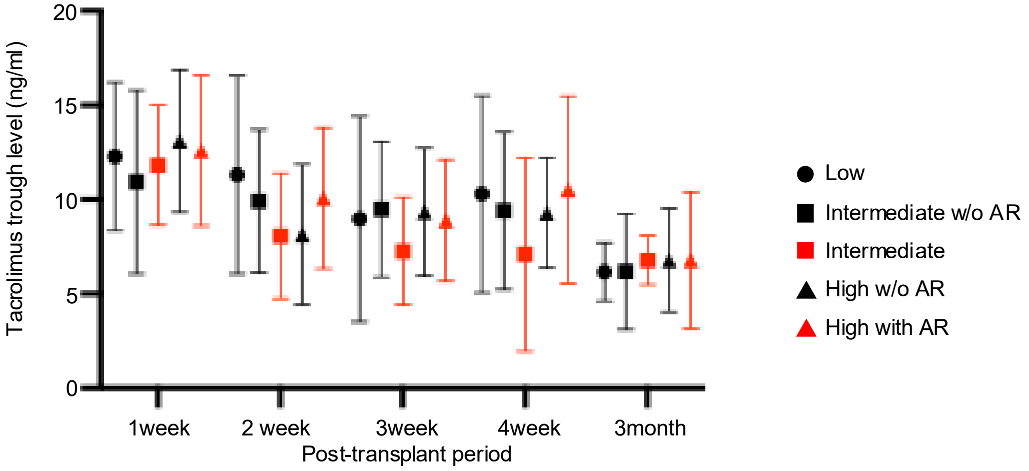

Supplementary Figure 11. Kinetics of tacrolimus trough levels after living-donor liver transplantation (LDLT).

Symbols and error bars indicate mean ± SD tacrolimus trough levels in the low (circles), intermediate (squares), and high (triangles) alloresponse groups, with or without acute rejection (red and black, respectively). Abbreviation: AR, acute rejection.

Supplementary Table 1. SIRPα genotype of healthy volunteer used for in vitro assays by Sanger sequence

Volunteer #	SIRPα Variant	Binding assay	Phenotype	T-cell assay
#1	V1/V1	○		
#2	V1/V1	○		
#3	V1/V1	○	○	
#4	V1/V1	○	○	
#5	V1/V1	○	○	○
#6	V1/V1	○		
#7	V1/V1	○		
#8	V1/V1	○		
#9	V1/V1		○	
#10	V1/V1	○		
#11	V1/V1	○		
#12	V2/V2	○		
#13	V2/V2	○		
#14	V2/V2	○		
#15	V2/V2	○		
#16	V2/V2	○		
#17	V2/V2	○	○	
#18	V2/V2	○	○	
#19	V2/V2	○	○	○
#20	V2/V2	○	○	○
#21	V2/V2		○	
#22	V2/V2		○	
#23	V2/V2		○	
#24	V2/V2		○	
#25	V2/V2			○
#26	V2/V2			○
#27	V2/V2	○		○
#28	V2/V2	○		○
#29	V2/V2	○		
#30	V2/V2	○		
#31	V1/V2			○
#32	V1/V2			○
#33	V1/V2			○
#34	V1/V2			○
#35	V1/V2			○
#36	V1/V2			○

Supplementary Table 2. The distribution of allele frequency of SIRPα variants in recipient and donor of living donor liver transplantation

Allele frequency	Recipients (n=154)	Donors (n=154)
V1, % (n)	35.4 (109)	36 (111)
V2, % (n)	63 (194)	61.7 (190)
V9, % (n)	1 (3)	1.6 (5)
NV1, % (n)	0.3 (1)	0.3 (1)
NV2, % (n)	0.3 (1)	0.3 (1)

Abbreviations: V, variant; NV, new variant

Supplementary Table 3. The primary disease of LDLT recipient was not associated with allele frequency of SIRPα polymorphism

Allele frequency	Viral (n=86)	Autoimmune (n=18)	Others (n=50)
V1, %	35.5	36.1	35
V2, %	63.3	63.9	62
V9, %	0.6	-	2
NV1, %	-	-	1
NV2, %	0.6	-	-

Abbreviations: V, variant; NV, new variant

Supplementary Table 4. Demographic characteristics of patients underwent living donor liver transplantation subgrouped by potential alloimmune response

Variables	Low-alloresponse group n=13	Intermediate-alloresponse group n=28	High-alloresponse group n=44	p value
Recipient age (year)	57 (32 - 69)	56 (19 - 70)	54 (26 - 69)	ns
Recipient gender male/ female	5 / 8	21 / 7	21 / 23	0.03
Sex match/ mismatch	6 / 7	10 / 18	21 / 23	ns
Relationship first degree/ others	13 / 0	19 / 9	32 / 12	0.02
ABO identical/ compatible/ incompatible	6 / 7 / 0	21 / 7 / 0	27 / 17 / 0	ns
Autoimmune disease -/+	11 / 2	26 / 2	39 / 5	ns
MELD score	17 (12 - 39)	18 (6 - 38)	22 (9 - 40)	ns
Donor age (year)	37 (24 - 64)	42 (24 - 66)	39 (21 - 63)	ns
Donor gender male/ female	10 / 3	17 / 11	26 / 18	ns
Number of HLA class I mismatch	2 (0 - 3)	2 (0 - 4)	2 (0 - 4)	ns
Number of HLA class II mismatch	1 (0 - 2)	1 (0 - 2)	1 (0 - 2)	ns
GRWR	0.91 (0.57 - 1.18)	0.83 (0.63 - 1.09)	0.92 (0.63 - 1.39)	ns
Closing portal pressure (mmHg)	16 (12 - 22)	18 (11 - 31)	17 (8 - 30)	ns
Total ischemic time (minute)	131 (72 - 210)	156 (65 - 673)	120 (54 - 273)	ns
Blood loss (ml)	3886 (500 - 14500)	5710 (810 - 23784)	5629 (880 - 19600)	ns
Operational time (minute)	775 (454 - 976)	792 (535 - 1311)	797 (550 - 1276)	ns

Numeric data are indicated as median (range). A difference was considered significant if the p-value was <0.05.
Abbreviations: MELD, model of end-stage liver disease; HLA, human leukocyte antigen; GRWR, graft-to-recipient weight ratio.

Supplementary Table 5. Adjusted variables in one-to-one pairwise propensity score matching analysis for patients in low- and intermediate-alloresponse groups

Variables	Low-alloresponse group n=8	Intermediate-alloresponse group n=8	p value
Recipient age (year)	59 (32 - 67)	61 (54 - 69)	ns
Recipient gender male/ female	5 / 3	5 / 3	ns
Relationship first degree/ others	8 / 0	8 / 0	ns
Autoimmune disease -/+	7 / 1	8 / 0	ns
Donor age (year)	37 (24 - 64)	31 (24 - 42)	ns
Donor gender male/ female	7 / 1	7 / 1	ns
Number of HLA class I mismatch	2 (0 - 2)	2 (1 - 3)	ns
Number of HLA class II mismatch	1 (0 - 1)	1 (0 - 1)	ns

Numeric data are indicated as median (range). A difference was considered significant if the p-value was <0.05.
Abbreviations: HLA, human leukocyte antigen;

Supplementary Table 6. Adjusted variables in one-to-one pairwise propensity score matching analysis for patients in low- and high-alloresponse groups

Variables	Low-alloresponse group n=13	High-alloresponse group n=13	p value
Recipient age (year)	57 (32 - 69)	57 (33 - 68)	ns
Recipient gender male/ female	5 / 8	6 / 7	ns
Relationship first degree/ others	13 / 0	13 / 0	ns
Autoimmune disease -/+	11 / 2	12 / 1	ns
Donor age (year)	37 (24 - 64)	37 (21 - 60)	ns
Donor gender male/ female	10 / 3	12 / 1	ns
Number of HLA class I mismatch	2 (0 - 3)	2 (0 - 2)	ns
Number of HLA class II mismatch	1 (0 - 2)	1 (0 - 1)	ns

Numeric data are indicated as median (range). A difference was considered significant if the p-value was <0.05.
Abbreviations: HLA, human leukocyte antigen;

Supplementary Table 7. Adjusted variables in one-to-one pairwise propensity score matching analysis for patients in intermediate- and high-alloresponse groups

Variables	Intermediate-alloresponse group n=23	High-alloresponse group n=23	p value
Recipient age (year)	55 (19 - 70)	56 (33 - 68)	ns
Recipient gender male/ female	16 / 7	16 / 7	ns
Relationship first degree/ others	15 / 8	16 / 7	ns
Autoimmune disease -/+	21 / 2	23 / 0	ns
Donor age (year)	41 (24 - 66)	39 (21 - 60)	ns
Donor gender male/ female	14 / 9	18 / 5	ns
Number of HLA class I mismatch	2 (0 - 4)	2 (0 - 4)	ns
Number of HLA class II mismatch	1 (0 - 2)	1 (0 - 2)	ns

Numeric data are indicated as median (range). A difference was considered significant if the p-value was <0.05.
Abbreviations: HLA, human leukocyte antigen;

Supplementary Table 8. Individual data of 85 patients underwent living donor liver transplantation

Patient#	Recipient age (year)	Recipient gender	Sex matching	Relationship	AB0 blood compatibility	Autoimmune disease	MELD score	Donor age (years)	Donor gender	Number of HLA class I mismatch	Number of HLA class II mismatch	GRWR	Closing portal pressure (mmHg)	Total ischemic time (minute)	Blood loss (ml)	Operational time (minute)	Recipient SIRPa Variant	Donor SIRPa Variant	Magnitude of presentation by recipient APC	Magnitude of presentation by donor APC	Sum of presentation magnitude	Potential alloresponse group	Acute Rejection (AR)	Chronic rejection (CR)	Billiary complication (BC)	Overall survival (years)	Outcome (alive/dead)
1	50	Male	Mismatch	Others	Identical	-	7	48	Female	3	2	0.91	13	132	4200	930	V1/V2	V1/V2	3	3	6	Intermediate	AR	CR	BC	7.0	dead
2	54	Male	Mismatch	Others	Identical	-	6	52	Female	4	2	0.98	17	106	1600	745	V2/V2	V1/V1	2	4	6	Intermediate	NO	NO	BC	16.0	alive
3	54	Male	Mismatch	Firstdegree	Identical	-	6	56	Female	1	1	0.90	12	101	1370	535	V1/V2	V1/V2	3	3	6	Intermediate	AR	NO	BC	15.4	alive
4	58	Female	Mismatch	Firstdegree	Compatible	-	40	28	Male	2	1	0.82	15	73	2500	550	V2/V2	V2/V2	4	4	8	High	NO	NO	NO	15.7	alive
5	65	Female	Mismatch	Firstdegree	Compatible	-	15	38	Male	1	1	0.57	22	94	2400	815	V1/V2	V1/V1	2	3	5	Low	NO	NA	NO	0.1	dead
6	54	Male	Match	Firstdegree	Identical	-	21	51	Male	2	1	0.73	25	151	5040	715	V1/V2	V1/V2	3	3	6	Intermediate	NO	NO	NO	3.3	dead
7	33	Female	Mismatch	Firstdegree	Identical	-	10	60	Male	2	1	0.98	30	273	9900	1205	V2/V2	V1/V2	3	4	7	High	AR	NO	NO	0.4	dead
8	55	Male	Mismatch	Firstdegree	Identical	-	22	29	Female	1	1	0.70	21	166	10200	752	V2/V2	V2/V2	4	4	8	High	NO	NO	NO	15.1	alive
9	36	Male	Match	Firstdegree	Compatible	+	19	31	Male	2	0	0.79	18	110	6000	889	V1/V2	V1/V2	3	3	6	Intermediate	AR	NA	NO	0.2	dead
10	59	Male	Mismatch	Firstdegree	Identical	-	38	30	Female	4	2	0.80	27	136	7000	823	V1/V2	V1/V2	3	3	6	Intermediate	AR	NO	NO	14.6	alive
11	57	Male	Match	Firstdegree	Identical	-	16	25	Male	2	1	0.82	15	172	4830	870	V1/V1	V1/V2	3	2	5	Low	NO	CR	BC	1.2	dead
12	69	Male	Match	Firstdegree	Identical	-	12	35	Male	2	1	1.12	15	131	14500	830	V1/V1	V1/V1	2	2	4	Low	NO	NO	BC	3.3	dead
13	60	Male	Mismatch	Firstdegree	Identical	-	6	33	Female	2	1	0.86	15	173	4200	735	V1/V2	V1/V2	3	3	6	Intermediate	NO	NO	NO	14.3	alive
14	66	Male	Mismatch	Firstdegree	Identical	-	19	60	Female	0	0	1.09	14	136	5610	755	V1/V2	V1/V2	3	3	6	Intermediate	NO	NO	BC	13.9	alive
15	58	Male	Match	Firstdegree	Compatible	-	31	28	Male	2	1	1.19	25	146	5400	753	V2/V2	V1/V2	3	4	7	High	NO	NO	BC	5.1	alive
16	36	Male	Mismatch	Others	Compatible	+	13	57	Female	1	0	1.33	16	147	4297	814	V2/V2	V1/V2	3	4	7	High	NO	NO	NO	4.2	alive
17	62	Female	Match	Firstdegree	Identical	-	15	37	Female	2	1	0.98	14	87	8780	1167	V2/V2	V1/V2	3	4	7	High	NO	NO	NO	14.1	alive
18	40	Female	Mismatch	Others	Identical	-	39	43	Male	4	2	1.39	8	77	2570	595	V2/V2	V1/V2	3	4	7	High	NO	CR	NO	0.4	dead
19	40	Male	Match	Firstdegree	Identical	-	26	43	Male	2	1	0.96	15	116	9700	934	V2/V2	V2/V2	4	4	8	High	AR	NO	NO	2.3	alive
20	58	Male	Match	Firstdegree	Compatible	-	13	29	Male	2	1	1.02	13	78	1280	690	V1/V1	V1/V1	2	2	4	Low	NO	NO	NO	0.3	dead
21	54	Male	Match	Firstdegree	Identical	-	12	25	Male	1	1	0.89	25	184	3650	877	V1/V2	V2/V2	4	3	7	High	AR	NO	BC	13.6	alive
22	52	Male	Mismatch	Firstdegree	Identical	-	36	46	Female	0	0	0.75	14	100	6640	768	V2/V2	V2/V2	4	4	8	High	NO	NO	BC	7.4	dead
23	58	Male	Match	Firstdegree	Identical	-	31	29	Male	0	0	0.75	15	75	6600	617	V2/V2	V1/V2	3	4	7	High	NO	NO	NO	13.4	alive
24	60	Male	Mismatch	Firstdegree	Compatible	-	33	25	Female	0	0	0.63	17	156	5100	947	V1/V2	V1/V2	3	3	6	Intermediate	AR	NO	NO	13.4	alive
25	58	Female	Mismatch	Others	Identical	-	13	64	Male	3	1	0.70	16	65	3300	610	V2/V2	V1/V1	2	4	6	Intermediate	AR	NA	NO	0.2	dead
26	29	Female	Match	Others	Identical	-	36	55	Female	3	1	1.23	8	146	1040	827	V2/V2	V2/V2	4	4	8	High	AR	NO	BC	7.6	dead
27	56	Female	Mismatch	Firstdegree	Identical	-	15	29	Male	2	1	0.74	17	144	2160	769	V1/V2	V1/V1	2	3	5	Low	NO	NO	NO	9.7	alive
28	61	Female	Mismatch	Others	Compatible	-	25	39	Male	2	1	0.87	16	66	4780	823	V2/V2	V1/V2	3	4	7	High	NO	NO	NO	9.5	alive
29	68	Female	Mismatch	Firstdegree	Compatible	-	9	45	Male	1	1	0.81	15	71	3800	630	V2/V2	V1/V2	3	4	7	High	AR	NA	NO	0.1	dead
30	57	Male	Mismatch	Others	Identical	-	37	56	Female	1	1	0.87	14	78	6190	819	V2/V2	V1/V2	3	4	7	High	NO	NO	NO	4.6	dead
31	69	Female	Match	Firstdegree	Identical	-	13	46	Female	1	1	1.00	15	77	2500	660	V2/V2	V2/V2	4	4	8	High	AR	NO	BC	0.6	dead
32	57	Male	Mismatch	Firstdegree	Compatible	-	13	27	Female	1	1	0.66	15	115	2800	738	V2/V2	V2/V2	4	4	8	High	NO	NO	NO	12.9	alive
33	52	Male	Mismatch	Others	Identical	-	11	48	Female	3	2	0.80	17	138	6400	749	V1/V1	V2/V2	4	2	6	Intermediate	NO	NO	BC	3.3	dead
34	51	Female	Match	Firstdegree	Identical	+	16	45	Female	2	1	0.76	14	121	1950	773	V2/V2	V1/V2	3	4	7	High	NO	NO	NO	8.3	dead
35	60	Female	Mismatch	Firstdegree	Identical	+	17	36	Male	2	1	0.97	16	102	11780	753	V2/V2	V1/V2	3	4	7	High	NO	NO	NO	11.3	dead
36	43	Female	Mismatch	Others	Identical	+	19	41	Male	4	2	0.75	15	95	3900	768	V2/V2	V2/V2	4	4	8	High	AR	NO	BC	11.0	dead
37	47	Male	Match	Others	Compatible	-	13	21	Male	1	0	0.76	23	93	8200	911	V2/V2	V2/V2	4	4	8	High	NO	NO	NO	12.4	alive
38	58	Male	Mismatch	Others	Compatible	-	11	57	Female	2	1	0.91	20	86	3170	676	V1/V2	V2/V2	4	3	7	High	AR	NO	BC	12.4	alive
39	26	Female	Mismatch	Others	Identical	-	33	24	Male	2	1	0.97	10	66	880	703	V2/V2	V2/V2	4	4	8	High	NO	NO	NO	12.3	alive
40	61	Female	Mismatch	Firstdegree	Identical	-	11	36	Male	1	0	0.83	14	54	1600	790	V1/V2	V2/V2	4	3	7	High	NO	NO	NO	9.0	alive
41	61	Male	Match	Firstdegree	Identical	-	22	26	Male	2	1	0.90	13	89	5160	710	V1/V2	V2/V2	4	3	7	High	NO	NA	NO	0.1	dead
42	56	Male	Match	Others	Identical	-	17	38	Male	4	2	0.80	12	78	5260	666	V1/V2	V1/V2	3	3	6	Intermediate	NO	NO	NO	12.2	alive
43	61	Male	Match	Firstdegree	Identical	-	24	33	Male	2	1	0.95	16	101	12520	976	V2/V2	V2/V2	4	4	8	High	NO	NO	NO	8.3	alive
44	65	Male	Match	Firstdegree	Identical	-	13	32	Male	1	0	0.77	16	109	6100	814	V1/V2	V1/V2	3	3	6	Intermediate	NO	NO	NO	11.6	alive
45	63	Male	Match	Firstdegree	Identical	-	11	29	Male	3	1	0.83	12	80	810	714	V1/V1	V2/V2	4	2	6	Intermediate	AR	NO	NO	11.6	alive
46	19	Female	Mismatch	Others	Compatible	-	6	53	Male	0	0	0.77	11	162	2800	854	V1/V2	V1/V2	3	3	6	Intermediate	NO	CR	NO	0.7	alive
47	64	Female	Mismatch	Firstdegree	Compatible	-	17	32	Male	1	1	1.04	17	137	4900	849	V1/V1	V1/V2	3	2	5	Low	NO	NO	BC	11.0	alive
48	61	Male	Mismatch	Others	Compatible	-	13	60	Female	4	2	0.83	9	135	3500	668	V2/V2	V2/V2	4	4	8	High	AR	NO	NO	0.9	dead
49	40	Female	Match	Firstdegree	Identical	-	27	43	Female	2	2	0.94	17	72	850	454	V1/V2	V1/V1	2	3	5	Low	NO	CR	NO	9.4	alive
50	68	Female	Mismatch	Firstdegree	Compatible	-	13	30	Male	1	1	1.04	13	80	4700	783	V1/V1	V1/V1	2	2	4	Low	NO	NO	NO	11.1	alive
51	55	Male	Match	Firstdegree	Compatible	-	13	30	Male	2	1	0.73	22	101	4500	749	V1/V2	V1/V2	3	3	6	Intermediate	AR	NO	NO	0.3	alive
52	68	Female	Match	Firstdegree	Identical	-	18	47	Female	2	1	1.26	16	83	1920	614	V2/V2	V2/V2	4	4	8	High	NO	NO	BC	11.0	alive
53	51	Male	Mismatch	Firstdegree	Compatible	-	34	25	Female	2	1	0.91	12	92	10300	774	V2/V2	V1/V2	3	4	7	High	NO	NO	NO	11.0	alive
54	54	Female	Match	Firstdegree	Compatible	-	10	28	Female	1	1	0.82	22	79	5870	717	V1/V2	V2/V2	4	3	7	High	NO	NO	NO	10.8	alive
55	58	Male	Mismatch	Firstdegree	Identical	-	15	28	Female	2	1	0.89	11	228	17200	1161	V1/V2	V1/V2	3	3	6	Intermediate	AR	NO	BC	10.6	alive
56	46	Female	Mismatch	Firstdegree	Identical	-	33	21	Male	2	1	0.95	13	87	3800	688	V2/V2	V2/V2	4	4	8	High	AR	NO	NO	10.5	alive
57	54	Female	Mismatch	Firstdegree	Identical	-	17	25	Male	2	0	0.89	11	91	3800	751	V2/V2	V2/V2	4	4	8	High	NO	NO	NO	0.6	alive
58	62	Male	Match	Firstdegree	Compatible	-	9	31	Male	1	1	0.66	13	95	3000	624	V2/V2	V2/V2	4	4	8	High	NO	NO	NO	10.5	alive
59	65	Female	Match	Firstdegree	Identical	-	29	41	Female	3	1	0.71	27	152	5200	1068	V1/V2	V2/V2	4	3	7	High	NO	NO	NO	10.0	alive
60	63	Male	Match	Firstdegree	Compatible	-	14	37	Male	2	1	1.18	18	172	3800	867	V1/V1	V1/V2	3	2	5	Low	NO	NO	BC	10.1	alive
61	53	Male	Match	Firstdegree	Identical	-	29	32	Male	1	1	0.95	18	264	19600	1276	V1/V2	V2/V2	4	3	7	High	NO	NO	NO	6.2	alive
62	67	Female	Mismatch	Firstdegree	Identical	+	12	44	Male	0	0	0.77	12	86	500	627	V1/V1	V1/V2	3	2	5	Low	NO	NO	NO	9.7	alive
63	41	Female	Match	Firstdegree	Identical	+	39	45	Female	3	2	0.83	17	194	2110	810	V1/V1	V1/V1	2	2	4	Low	NO	NA	NO	0.2	dead
64	32	Female	Mismatch	Firstdegree	Identical	-	14	64	Male	2	1	1.01	17	134	2530	740	V1/V1	V1/V2	3	2	5	Low	NO	NO	NO	9.0	alive
65	66	Female	Mismatch	Firstdegree	Identical	-	29	37	Male	2	1	0.73	15	122	820	629	V1/V2	V1/V2	3	3	6	Intermediate	NO	NO	BC	5.2	alive
66	28	Female	Match	Firstdegree	Identical	-	39	63	Female	2	1	0.83	18	172	1750	794	V2/V2	V2/V2	4	4	8	High	AR	CR	NO	9.9	alive
67	66	Male	Match	Firstdegree	Identical	-	16	37	Male	2	1	0.93	13	165	9966	829	V1/V2	V1/V2	3	3	6	Intermediate	NO	NO	NO	8.3	alive
68	54	Male	Match	Firstdegree	Identical	-	28	26	Male	1	1	0.89	18	127	8690	743	V1/V2	V1/V2	3	3	6	Intermediate	NO	NO	NO	0.4	dead
69	50	Male	Match	Firstdegree	Compatible	-	19	54	Male	1	1	0.75	25	117	3580	692	V1/V2	V1/V2	3	3	6	Intermediate	AR	NA	NO	0.2	dead
70	61	Female	Match	Firstdegree	Compatible	+	24	57	Female	0	0	1.03	19	118	3924	732	V2/V2	V1/V2	3	4	7	High	NO	NA	NO	0.2	dead
71	56	Male	Mismatch	Others	Identical	-	20	51	Female	4	2	1.23	20	127	11713	919	V1/V2	V2/V2	4	3	7	High	NO	NO	BC	7.5	alive
72	69	Male	Mismatch	Firstdegree	Compatible	-	6	40	Female	1	1	0.73	13	115	2966	740	V1/V2	V1/V2	3	3	6	Intermediate	NO	NO	BC	7.4	alive
73	59	Male	Mismatch	Firstdegree	Compatible	-	14	24	Female	2	0	0.82	19	210	5964	976	V1/V2	V1/V1	2	3	5	Low	NO	NO	NO	7.5	alive
74	64	Female	Mismatch	Firstdegree	Compatible	-	29	39	Male	2	1	0.99	22	98	4041	647	V1/V2	V2/V2	4	3	7	High	NO	NO	NO	0.5	dead
75	66	Female	Mismatch	Others	Compatible	-	21	66	Male	4	2	0.91	25	142	4470	799	V1/V2	V1/V2	3	3	6	Intermediate	AR	NA	NO	0.1	dead
76	47	Male	Match	Firstdegree	Compatible	-	29	21	Male	0	0	0.69	27	206	13195	923	V1/V2	V2/V2	4	3	7	High	NO	NO	BC	6.5	alive
77	19	Male	Match	Others	Identical	-	29	45	Male	2	2	0.79	24	101	2786	625	V1/V2	V1/V2	3	3	6	Intermediate	NO	NA	NO	0.0	dead
78	58	Female	Mismatch	Others	Identical	+	16	51	Male	3	2	0.99	31	673	23784	1311	V1/V1	V2/V2	4	2	6	Intermediate	NO	NA	NO	0.0	dead
79	66	Female	Mismatch	Firstdegree	Compatible	-	17	37	Male	2	1	1.07	23	121	2984	641	V2/V2	V1/V2	3	4	7	High	AR	NO	NO	4.7	alive
80	69	Female	Mismatch	Firstdegree	Identical	-	24	42	Male	2	1	0.98	19	158	1100	605	V1/V2	V1/V2	3	3	6	Intermediate	NO	NA	NO	0.1	dead
81	56	Female	Mismatch	Others	Compatible	-	10	59	Male	4	2	0.75	18	178	4320	843	V1/V2	V2/V2	4	3	7	High	AR	NO	NO	3.5	alive
82	54	Female	Mismatch	Firstdegree	Identical	-	25	24	Male	2	0	0.79	23	235	9578	973	V1/V2	V1/V2	3	3	6	Intermediate	AR	NO	BC	2.9	alive
83	66	Male	Match	Firstdegree	Identical	-	13	31	Male	2	1	0.63	14	243	2232	920	V2/V2	V2/V2	4	4	8	High	AR	NO	NO	2.9	alive
84	64	Male	Match	Firstdegree	Identical	-	9	31	Male	1	1	1.33	26	147	6041	900	V2/V2	V1/V2	3	4	7	High	NO	NO	BC	1.8	alive
85	70	Male	Mismatch	Firstdegree	Identical	-	38	41	Female	1	1	0.80	25	240	5642	844	V1/V2	V1/V2	3	3	6	Intermediate	AR	NA	NO	0.0	dead

Supplementary Table 9. Mycophenolate mofetil use after living-donor liver transplantation.

	**Low-alloresponse group (n=13)**	**Intermediate-alloresponse group (n=28)**	**High-alloresponse group (n=44)**	**P value**
MMF administration, n (%)	7 (53.8%)	20 (71.4%)	22 (50.0%)	ns
Maintenance dose of MMF (range, mg/body/day)	500-1000	500-1000	500-1000	-

Abbreviations: MMF, Mycophenolate Mofetil

Supplementary Table 10. Rejection Activity Index (RAI) for biopsy-proven rejection (Banff 1997 criteria).

	Portal inflammation	Bile duct inflammation	Venous endothelial inflammation	Severity
BPR 1	2	2	1	Moderate
BPR 2	1	1	1	Mild
BPR 3	2	2	3	Severe
BPR 4	1	1	1	Mild
BPR 5	1	1	1	Mild

Abbreviations: BPR, Biopsy-proven rejection

Supplementary material information: Company provided sequence information of recombinant SIRPa-Fc V1 and V2 proteins highly polymorphic IgV-domain.

Protein (cat#)	Company	Aminoacid sequence
SIRPa-Fc (V1) (786506)	Biolegend	EEELQVIQPDKSVLVAAGETATLRCTATSLIPVGPIQWFRGAGPGRELIYNQKEG HFPRVTTVSDLTKRNNMDFSIRIGNITPADAGTYYCVKFRKGSPDDVEFKSGAG TELSVRA
SIRPa-Fc (V2) (LS-G40300-50)	LsBio	EEELQVIQPDKSVSVAAGESAILHCTVTSLIPVGPIQWFRGAGPARELIYNQKEG HFPRVTTVSESTKRENMDFSISISNITPADAGTYYCVKFRKGSPD-TEFKSGAGTELSVRA
